# Human ancestry correlates with language and reveals that race is not an objective genomic classifier

**DOI:** 10.1038/s41598-017-01837-7

**Published:** 2017-05-08

**Authors:** Jennifer L. Baker, Charles N. Rotimi, Daniel Shriner

**Affiliations:** Center for Research on Genomics and Global Health, National Human Genome Research Institute, Building 12A, Room 4047, 12 South Drive, Bethesda, Maryland, 20892 USA

## Abstract

Genetic and archaeological studies have established a sub-Saharan African origin for anatomically modern humans with subsequent migrations out of Africa. Using the largest multi-locus data set known to date, we investigated genetic differentiation of early modern humans, human admixture and migration events, and relationships among ancestries and language groups. We compiled publicly available genome-wide genotype data on 5,966 individuals from 282 global samples, representing 30 primary language families. The best evidence supports 21 ancestries that delineate genetic structure of present-day human populations. Independent of self-identified ethno-linguistic labels, the vast majority (97.3%) of individuals have mixed ancestry, with evidence of multiple ancestries in 96.8% of samples and on all continents. The data indicate that continents, ethno-linguistic groups, races, ethnicities, and individuals all show substantial ancestral heterogeneity. We estimated correlation coefficients ranging from 0.522 to 0.962 between ancestries and language families or branches. Ancestry data support the grouping of Kwadi-Khoe, Kx’a, and Tuu languages, support the exclusion of Omotic languages from the Afroasiatic language family, and do not support the proposed Dené-Yeniseian language family as a genetically valid grouping. Ancestry data yield insight into a deeper past than linguistic data can, while linguistic data provide clarity to ancestry data.

## Introduction

It is now possible to trace the migratory paths of anatomically modern humans using genetic data. Early research pointed to a sub-Saharan African origin for modern humans by around 200,000–150,000 years ago^1^, and analyses of autosomal markers^2^ and Y DNA haplogroups^3, 4^ suggest the earliest structuring of the human population occurred approximately 140,000 years ago^5–8^. Initial efforts to characterize the movement of early humans in relation to ancestry grouped populations according to five geographical regions: Sub-Saharan Africa, Europe/the Middle East/Central Asia/South Asia, East Asia, Oceania, and the Americas^9^. Subsequent analyses allowed for refinement of the genetic history of global ancestries, revealing regional structure through the identification of 7^10^, 14^11^, and 19 ancestries^2^.

Due to shared history, genetic and linguistic processes are expected to show congruent patterns of differentiation^12^. Two major ways to disrupt this congruence are gene flow and language replacement^12^. Prior research was limited by the numbers of loci and samples and did not account for admixture^13, 14^. By focusing on underlying ancestries rather than samples, confounding due to recent admixture is removed. We hypothesize that focusing on language families or branches, rather than languages, will mitigate problems arising from areal features and will provide a similar deeper level of resolution.

Here, we present the results of the largest-to-date global analysis of ancestry from 282 samples^10, 15–36^, providing greater resolution of worldwide ancestry and increasing the estimate of ancestries from 19 to 21^2^. Using a graph-based model of gene flow to estimate migration events from ancestry-specific allele frequencies^37^, we find evidence for migration events in the distant past. These abundant genomic data provide an exciting opportunity to test linguistic hypotheses involving multiple language families. Conversely, the linguistic data help resolve inconsistencies observed in the genomic data. Consistent with prior findings^2, 11^, ancestral heterogeneity is observed in the vast majority of individuals and samples and on all continents, as well as in racial and ethnic groups.

## Results

### Admixture analysis

We merged genotype data from 282 samples from 23 regional and global diversity projects, yielding a total of 5,966 individuals and 19,075 SNPs (Table S1). To address the possible effect of SNP ascertainment bias on *F*_*ST*_ estimation, we compared pairwise estimates for the 26 samples from the 1000 Genomes Project^31^ based on our panel of genotyped SNPs *vs*. the whole genome sequences. The median difference was 0.0030 (95% confidence interval [−0.0002, 0.0177]), indicating that *F*_*ST*_ estimation was not significantly biased by SNP ascertainment or the size of our panel of SNPs.

**Figure 1:**
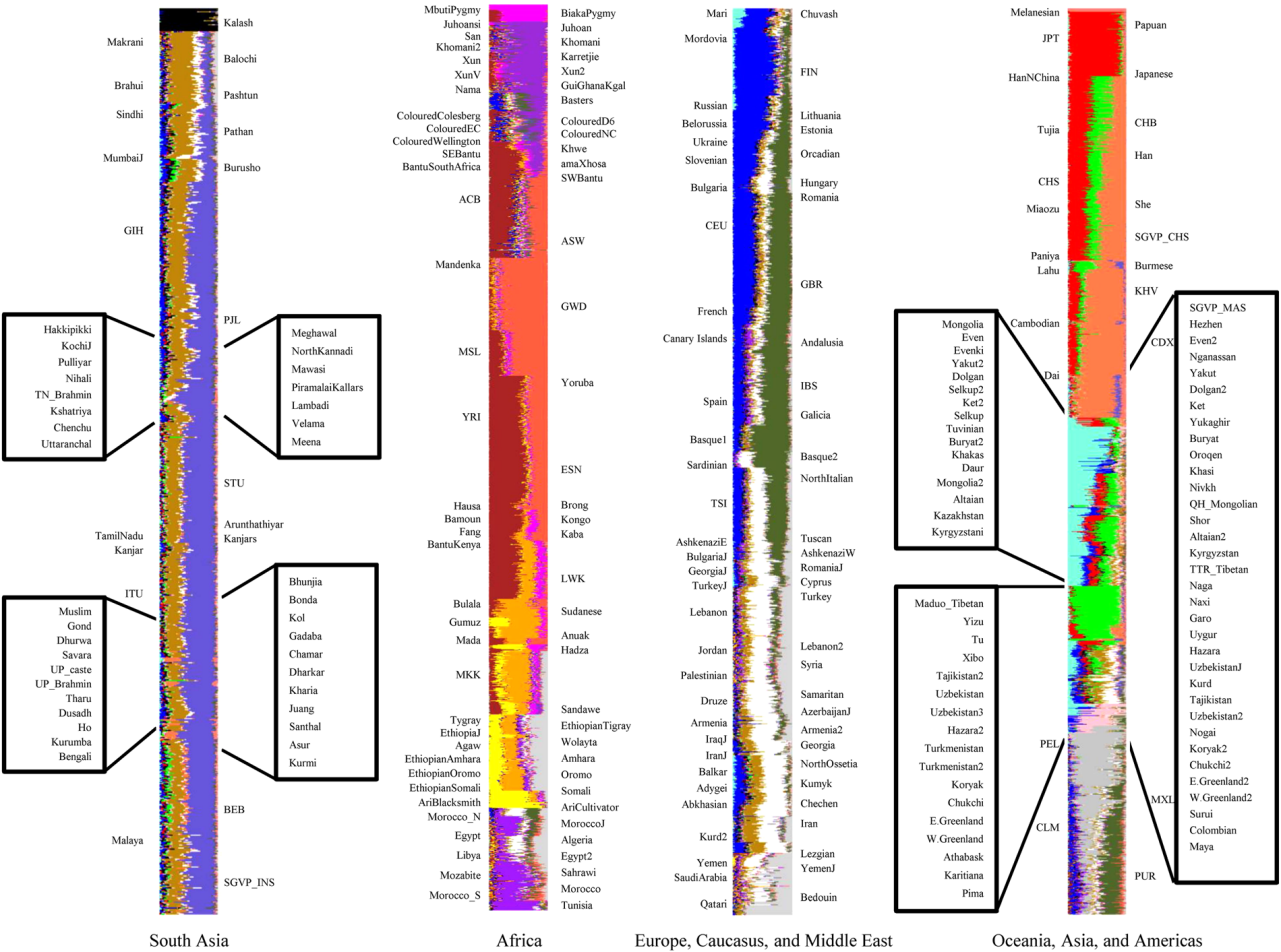
Ancestry analysis of the global data set. The 282 samples are labeled alternating in the left and right margins. The 21 ancestral components are Kalash (black), Southern Asian (dark goldenrod), South Indian (slate blue), Central African (magenta), Southern African (dark orchid), West-Central African (brown), Western African (tomato), Eastern African (orange), Omotic (yellow), Northern African (purple), Northern European (blue), Southern European (dark olive green), Western Asian (white), Arabian (light gray), Oceanian (salmon), Japanese (red), Southeastern Asian (coral), Northern Asian (aquamarine), Sino-Tibetan (green), Circumpolar (pink), and Amerindian (gray).

Unsupervised clustering yielded support for 21 subcontinental ancestries (Fig. 1 and Fig. S1). The posterior mode of *K* was also 21, with a 100% highest posterior density interval [18, 23]. Of the 21 ancestries, 18 were previously observed^2^. The only previously observed ancestry not present in this set of 21 was ancestry predominantly found in Cushitic-speaking peoples from East Africa, which we subsequently refer to in shorthand as Cushitic ancestry. Given that Cushitic ancestry has been detected before^2, 11^, its absence in the current data set indicates a need of additional sampling for proper classification. Our analysis identified three new ancestries: (1) Western African, (2) Circumpolar, and (3) Southern Asian. Our data support the hypothesis that subcontinental geography is a strong proxy for ancestry (Fig. S2). Consequently, we labeled the 21 ancestries on the basis of present-day geographic distributions. The samples that are the best proxies for these ancestries are provided in Table S2 and the mixing proportions of all ancestries for all samples are provided in Table S3. Pairwise *F*_*ST*_ estimates between ancestries are provided in Table S4.

To investigate the stability of the ancestries, we tested the null hypothesis that no genetic differentiation exists between the previous and current definitions for each ancestry. First, we used Mantel’s test to assess the correlation between the *F*_*ST*_ matrix generated with ancestries as defined in this study compared to the one generated with ancestries as previously defined^2^. The matrices were matched by eliminating the three new ancestries from the current matrix and the Cushitic entry from the previous matrix, resulting in a comparison of two 18 × 18 matrices. The estimated correlation coefficient *r* = 0.992 was significantly different from *ρ* = 0 (1.28 × 10^−34^ ≤ *p* ≤ 2.56 × 10^−5^) but not significantly different from *ρ* = 1 (0.122 ≤ *p* ≤ 0.596), providing evidence for the overall stability of the clusters. Second, we tested whether *F*_*ST*_ was 0 for each of the 18 pairwise comparisons. For 14 ancestries, the previous and current definitions were not significantly different (Table S5). For Southeastern Asian, Sino-Tibetan, Western Asian, and South Indian ancestries, the differences were statistically significant, with changes in *F*_*ST*_ ranging from 0.010 to 0.021 (Table S5). Thus, seemingly small changes in the overall cross-validation score do not preclude significant changes in the allele frequency profiles of a subset of ancestries.

We next investigated the extent of ancestral heterogeneity throughout the hierarchy of population structure. First, we found that individuals with mixed ancestry were present on all continents (Fig. S2). Second, mixed ancestry was present in 96.8% of samples (Table S3), with a median of 6 ancestries per sample (95% confidence interval [1, 12]). To illustrate, the GBR (British in England and Scotland) sample had a mixture of 38.1% Northern European and 42.8% Southern European ancestries, with small but significant contributions from seven additional ancestries (Table S3). In the ACB sample (African Caribbeans in Barbados), “African” encompassed six ancestries and “European” encompassed four ancestries (Table S3). Similarly, the ASW sample (People with African ancestry in Southwest USA) included all 10 of these ancestries plus one additional ancestry to account for a Native American component (Table S3). The PUR sample (Puerto Ricans in Puerto Rico) had 13 ancestries. Third, consistent with earlier reports^2, 11^, mixed ancestry was present in 97.3% of individuals, with a median of 4 ancestries per individual (95% confidence interval [1, 7]).

### Migration events

**Figure 2:**
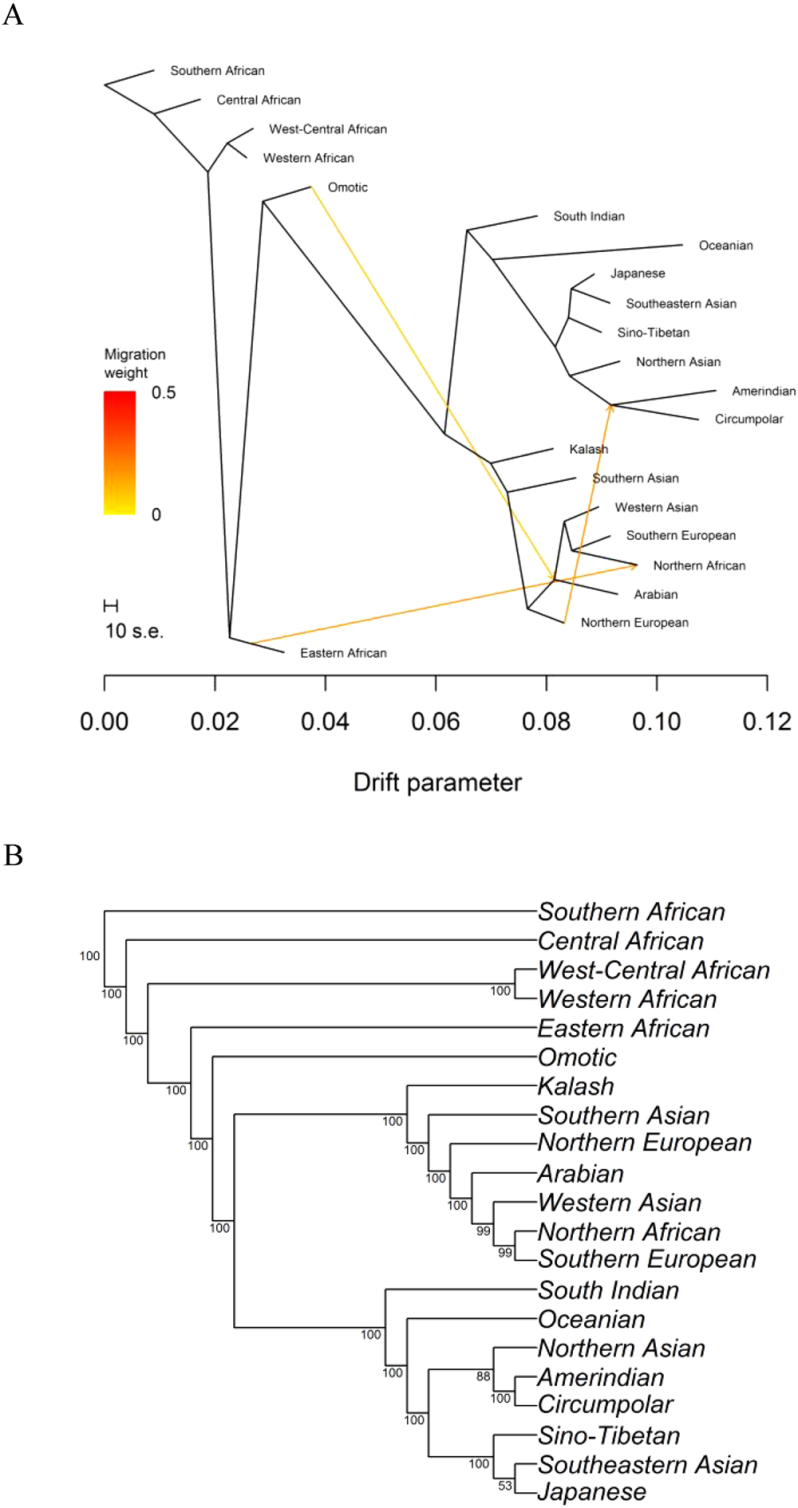
(**A**) The migration graph. TreeMix analysis suggests that migration events occurred between (1) Eastern African and Northern African ancestries; (2) Omotic ancestry and the node leading to Arabian, Northern African, Southern European, and Western Asian ancestries; and (3) Northern European ancestry and the node leading to Amerindian and Circumpolar ancestries. (**B**) Majority-rule consensus tree. The migration events were suppressed to emphasize the underlying topology.

We used TreeMix^37^ to infer the patterns of population splits and mixtures in the evolutionary history of the 21 ancestries. By analyzing ancestries instead of samples, the underlying model infers the structure of an ancestral population by linking modern ancestries to a common ancestor using ancestry-specific allele frequencies with the effects of recent admixture removed. This analysis revealed three migration events (Fig. 2). One migration event was between Eastern African and Northern African ancestries. This event is supported by the fact that E1b1b1b1a (formerly known as E-M81), the most common Y DNA haplogroup in North Africa, is a descendent of E1b1b, commonly found in Eastern Africa^38^. Another migration event was between Omotic ancestry and the node leading to Arabian, Northern African, Southern European, and Western Asian ancestries. We did not detect either of these two events previously^39^. When we added the previously defined Cushitic ancestry to the current set, we did not observe either event, suggesting that both events reflected the absence of Cushitic ancestry. The third migration event, which we did observe previously, was between Northern European and Amerindian ancestries. The identification of Circumpolar ancestry resulted in the migration edge moving from the terminal tip of Amerindian ancestry to the common ancestor of Amerindian and Circumpolar ancestries.

Three previously observed migration events^39^ were not evident in the current analysis. One, we did not observe an event between Arabian and Cushitic ancestries, because Cushitic ancestry was not present in the current data set. When we integrated the previously defined Cushitic ancestry into the current set, TreeMix grouped Cushitic ancestry with Eastern African and Omotic ancestries and inferred a migration event between Arabian and Cushitic ancestries, consistent with our previous results. Furthermore, Arabian, Eastern African, and Omotic ancestries were not significantly different in the presence or absence of Cushitic ancestry (Table S5). Taken together, these results support the hypothesis that Cushitic ancestry was formed by a mixture event. Two, we previously observed an inferred migration event between Indian and Arabian ancestries. Indian ancestry experienced the largest amount of redefinition with the additional data, whereas Arabian ancestry did not differ (Table S5). When we replaced the previous definition of Indian ancestry with the current one, no migration event was inferred. This result suggests that the original inference of a migration event reflected an underdefined Indian ancestry. Three, we previously observed an event involving Kalash and Northern European ancestries. Kalash ancestry was not significantly different between the two data sets (Table S3). When we added the newly defined Southern Asian ancestry, we observed the Kalash-Northern European event when Kalash ancestry was not grouped in the subtree with Southern Asian ancestry (36% of runs) but not when Kalash ancestry was grouped in the subtree with Southern Asian ancestry (64% of runs).

### Language

We were able to annotate 249 samples with language (Table S1). Our data set covers an estimated 21.3% of the 141 primary language families but 97.8% of people^40^. By focusing on ancestries rather than samples, confounding due to recent admixture is removed. We therefore evaluated correlations among ancestries and languages (Table S6).

**Figure 3:**
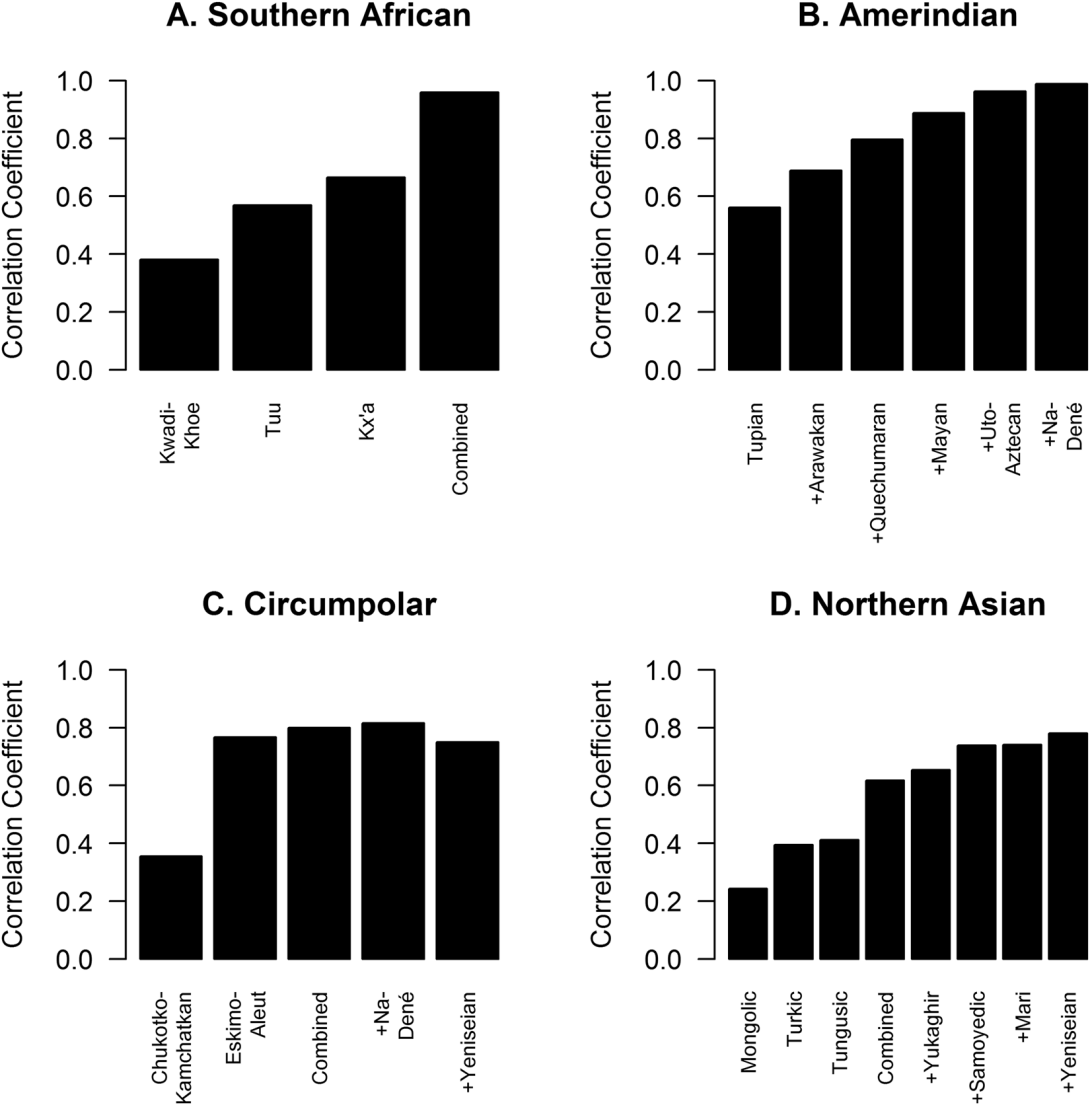
Correlation of ancestry and language. (**A**) “Combined” refers to Kwadi-Khoe, Tuu, and Kx’a, previously referred to collectively as Khoisan. (**B**) “+” indicates the combination of the listed language plus all languages listed to the left. Tupian, Arawakan, Quechumaran, Mayan, and Uto-Aztecan are referred to collectively as Amerind. (**C**) “Combined” refers to Chukotko-Kamchatkan and Eskimo-Aleut, referred to collectively as Paleo-Siberian. Note that inclusion of Yeniseian worsens the correlation. (**D**) “Combined” refers to Mongolic, Turkic, and Tungusic, referred to collectively as Altaic.

Southern African ancestry correlates with Kwadi-Khoe, Kx’a, and Tuu languages (*r* = 0.960, *p* = 4.78 × 10^−138^, Fig. 3A). Central African ancestry corresponds to Pygmies, both Eastern and Western (Table S3). Pygmies are thought to have lost their original language and now speak Niger-Congo or Nilo-Saharan languages, presumably adopted from neighboring tribes^41^. Consequently, Central African ancestry does not meaningfully correlate with extant language families.

Eastern African ancestry correlates with the Nilo-Saharan language family (*r* = 0.715, *p* = 2.39 × 10^−40^). Arabian ancestry correlates with the Semitic branch of the Afroasiatic language family (*r* = 0.774, *p* = 7.28 × 10^−51^). The Cushitic branch of the Afroasiatic language family correlates with both Eastern African (*r* = 0.417, *p* = 7.17 × 10^−12^) and Arabian (*r* = 0.336, *p* = 5.46 × 10^−8^) ancestries. This result is consistent with our previous finding that Cushitic ancestry formed by admixture between Nilo-Saharan and Arabian ancestries^39^. West-Central African ancestry correlates with both Bantu and non-Bantu languages in the Niger-Congo language family (*r* = 0.895, *p* = 2.00 × 10^−88^), whereas Western African ancestry correlates with Mande languages (*r* = 0.797, *p* = 5.64 × 10^−56^). West-Central and Western African ancestries are sibling ancestries (Fig. 2), but this result does not indicate whether Mande languages should be considered as part of the Niger-Congo language family.

Northern African ancestry correlates with the Berber branch of the Afroasiatic language family (*r* = 0.946, *p* = 1.48 × 10^−122^). Arabian and Northern African ancestries are both descended from the lineage that includes all Out of Africa migrants, whereas Omotic ancestry is descended from the lineage that includes all sub-Saharan ancestries (Fig. 2). Omotic ancestry correlates with the Omotic languages (*r* = 0.777, *p* = 1.40 × 10^−51^). Thus, the genomic data support the linguistic hypothesis that the Omotic languages are not part of the Afroasiatic family^42^.

Amerindian ancestry correlates with Tupian, Arawakan, Quechumaran, Mayan, and Uto-Aztecan languages (*r* = 0.962, *p* = 6.17 × 10^−142^, Fig. 3B), consistent with the hypothesized grouping of all these languages in the Amerind family^43^. Circumpolar ancestry correlates with both the Eskimo-Aleut and Chukotko-Kamchatkan language families (*r* = 0.799, *p* = 1.41 × 10^−56^, Fig. 3C), which collectively are known as Paleo-Siberian languages. The Athabask sample showed 64% Amerindian, 34% Circumpolar and 2% Northern Asian ancestry; accordingly, the Na-Dené language correlates with both Amerindian and Circumpolar ancestries but not with Northern Asian ancestry. Northern Asian ancestry correlates with Mongolic, Turkic, and Tungusic languages (*r* = 0.617, *p* = 1.53 × 10^−27^), which have been grouped into the Altaic language family. Additionally, Northern Asian ancestry correlates with the Samoyedic branch of the Uralic family, Yukaghir languages, the Mari language isolate, and Yeniseian languages (*r* = 0.781, *p* = 2.53 × 10^−52^, Fig. 3D).

Southern European ancestry correlates with both Italic and Basque speakers (*r* = 0.764, *p* = 6.34 × 10^−49^). Northern European ancestry correlates with Germanic and Balto-Slavic branches of the Indo-European language family as well as Finno-Ugric and Mordvinic languages of the Uralic family (*r* = 0.672, *p* = 4.67 × 10^−34^). Italic, Germanic, and Balto-Slavic are all branches of the Indo-European language family, while the correlation with languages of the Uralic family is consistent with an ancient migration event from Northern Asia into Northern Europe^39^. Kalash ancestry is widely spread but is the majority ancestry only in the Kalash people (Table S3). The Kalasha language is classified within the Indo-Iranian branch of the Indo-European language family.

South Indian ancestry correlates with the Dravidian language family, the Munda branch of the Austroasiatic language family, and Nihali, which has been alternatively classified as part of the Munda branch or as an isolate (*r* = 0.740, *p* = 2.03 × 10^−44^). Southern Asian ancestry correlates with the Indo-Iranian branch of the Indo-European language family as well as the Dravidian language family (*r* = 0.678, *p* = 7.96 × 10^−35^). Sino-Tibetan ancestry correlates with the Sino-Tibetan language family as well as with Monguor and Mongolic (*r* = 0.793, *p* = 3.83 × 10^−55^). Southeastern Asian ancestry correlates with the Mon-Khmer branch (specifically, Khmer and Vietic but not Khasi languages) of the Austroasiatic language family, the Tai-Kadai language family, and the Hmong-Mien language family (*r* = 0.686, *p* = 5.36 × 10^−36^). Japanese ancestry correlates with the Japonic language family (*r* = 0.644, *p* = 1.55 × 10^−30^). Oceanian ancestry correlates with the Austronesian and Papuan language families (*r* = 0.954, *p* = 3.36 × 10^−131^). Western Asian ancestry correlates with Northeast Caucasian, Northwest Caucasian, and Kartvelian language families as well as the Armenian branch of the Indo-European language family (*r* = 0.522, *p* = 831 × 10^−19^).

## Discussion

We have compiled and analyzed the largest available global data set of genotyped samples annotated with language. We found that additional sampling revealed three previously unknown ancestries. Due to sparse or nonexistent sampling in some parts of the world, there may be ancestries that remain unidentified. The finding that the vast majority of people have mixed ancestry^2, 11^ has been confirmed and extended. Importantly, mixed ancestry at the sample level does not reflect population stratification, *i*.*e*., two or more subsets of individuals ancestrally homogeneous within subsets and ancestrally heterogeneous between subsets, but rather reflects mixed ancestry at the individual level.

The labels ancestry, continent, ethno-linguistic group, ethnicity, and race have different ontological bases. Ancestry is determined solely by genomic data and is not subjectively self-identified. Being defined by DNA, ancestries are subject to evolutionary change, *i*.*e*., ancestries are subject to birth-death cycles and ancestry-specific allele frequencies can change over time. Ancestries are related through a phylogeny which describes ancestral and descendent relationships. As such, it is appropriate to ask how many ancestries existed at a specified period of time and what the ancestry-specific allele frequencies were at that time. Over the timespan of anatomically modern humans, most ancestries emerged after the Out-of-Africa migrations and no ancestries are near fixation. Almost no samples are ancestrally homogeneous; taken together, these findings indicate that ancestries should not be thought of as types. However, during peopling of the world, ancestries remained distinct long enough to acquire correlation with language.

Whereas the label ancestry is genomically defined, the label continent is geographically defined and the label ethno-linguistic group is socio-culturally defined. According to the United States Census, race and ethnicity are different constructs. Biological race is phenotypically defined, being based on a small set of physical characteristics^44^. However, in the 2010 US Census, there were 15 race categories, including several national-origin groups that are generally not considered to be races^45^. The category ethnicity was limited to either “Hispanic, Latino, or Spanish” or not, with the option of distinguishing nationality, *i*.*e*., Mexico, Puerto Rico, Cuba, or other^45^. Our data show that continent, ethno-linguistic group, race, and ethnicity all harbor substantial ancestral heterogeneity.

The group label race has a controversial history filled with alternative definitions and debates whether race is biologically real or a social construct^46–48^. Two lines of genetic evidence have been used to support the social construct position. One, apportionment of genetic variance into hierarchical groups relies on arbitrary thresholds and leads to incoherent classification^49, 50^. Two, the description of human genomic variation as clustered has led some to equate ancestry with continent and hence with race and has been countered with the argument that variation is clinal^50^. Our findings indicate that ancestry cross-classifies ethno-linguistic group as well as continent and race. To expound this point, Western Asian ancestry currently exists at its highest frequency in peoples from the Caucasus Mountains and the Levant and is the major ancestry in Abkhazian, Georgian, and Druze samples. Yet, significant amounts of Western Asian ancestry are present in samples with origins ranging from Morocco to Mongolia and from England to Ethiopia. That is, Western Asian ancestry simultaneously exists in Africa, Asia, and Europe, as well as in the US racial categories Black or African American, Asian, and White. Thus, in contrast to race, ancestry is a valid genomic classifier.

To illustrate the distinctions among these group labels, we provide two examples from genetic epidemiology. First, controlling for population structure in genome-wide association studies is necessary to prevent spurious association. One motivation for the use of principal components analysis to control for population structure was the spurious association of a SNP in the lactase gene *LCT* with height in European Americans due to an axis of variation that reflected differential ancestry from north to south Europe^51^. The racial label White fails to capture this difference in proportions of Northern *vs*. Southern European ancestry. Second, admixture mapping is a technique for mapping loci conferring differential risk by ancestry^52^. As applied to admixed African Americans, admixture mapping relies on genetic differentiation between ancestries from Africa and Europe. Uniformly classifying admixed African Americans with the racial label Black fails to capture inter-continental admixture and precludes use of the technique. Similarly, the SEBantu sample comprises individuals from the Sotho-Tswana and Zulu ethno-linguistic groups and reflects intra-continental admixture between Bantu-speaking peoples and indigenous Khoisan-speaking peoples. Uniformly classifying this sample with the continental label African or the racial label Black fails to capture the essential ancestral differences.

Migrations inferred by TreeMix reflect excess covariance and appear to reflect two types of situations. Most ancestries were formed by a splitting process; however, ancestry characteristic of Cushitic-speaking peoples was formed by a mixture process involving Eastern African and Arabian ancestries^39^. Consistent with admixture, Cushitic ancestry can be either grouped with Eastern African ancestry and connected by migration to Arabian ancestry or grouped with Arabian ancestry and connected by migration to Eastern African ancestry^39^. Some inferred migration events reflect missing ancestries and/or underdefined ancestries, indicating the importance of dense sampling. Taken together, the results of the migration analyses are largely consistent with the serial founder model^10^, coupled with a low level of admixture.

Historical linguistics is considered to have an upper limit of ~10,000 years^53^. The ability of genomics to probe into the more distant past, combined with the correlation of ancestry and language, provides an opportunity to investigate historical linguistics on a deeper time scale. It remains unclear whether population structure provided a substrate for subsequent linguistic differentiation or whether language was a barrier to gene flow. In either case, we find moderate to strong correlations between ancestries and languages at the family or branch levels, providing evidence for and against several phylolinguistic hypotheses. One, Kwadi-Khoe, Kx’a, and Tuu languages previously were classified as the Central, Northern, and Southern branches, respectively, of the Khoisan family. Although this family classification is presently considered obsolete by many linguists^54^, our results provide genomic support for the validity of grouping these languages into one primary language family. Two, the common ancestor of West-Central African and Western African ancestries is a sibling to Eastern African ancestry (Fig. 2), consistent with the phylolinguistic hypothesis that the Niger-Congo and Nilo-Saharan language families are descended from a common ancestor called Kongo-Saharan^55^. Three, the correlation of Northern European ancestry with branches of Indo-European and Uralic language families suggests that additional Northern European samples may split Northern European ancestry into two ancestries, one corresponding to Indo-European speakers and the other corresponding to Finno-Ugric speakers of the Uralic family, distinct from Samoyedic speakers from the Uralic family. Four, Arabian and Northern African ancestries belong to the Out-of-Africa lineage, whereas Omotic ancestry clusters with sub-Saharan ancestries (Fig. 2). In turn, our results imply that Semitic and Berber languages, but not Omotic languages, correlate with the Out-of-Africa lineage. Furthermore, the Arabian parentage of Cushitic ancestry supports a Middle Eastern origin with a backflow to Eastern African, raising the possibility that Cushitic languages similarly have a Middle Eastern rather than an Eastern African origin, at least in part. Despite the notable absence of genomic data corresponding to Egyptian and Chadic languages, these results do not support the inclusion of Omotic languages in the Afroasiatic language family and are consistent with the hypothesis that that the Afroasiatic language family has a Middle Eastern origin. Additionally, we hypothesize that the migration events between Eastern African and Northern African ancestries as well as between Omotic ancestry and the node leading to Arabian, Northern African, Southern European, and Western Asian ancestries reflect excess covariance due to the absence of a distinct Cushitic ancestry. Five, our results provide resolution to “Ancestral South Indians” and “Ancestral North Indians”^56^. In particular, the prevalence of Y DNA haplogroup H in South India compared to the prevalence of Y DNA haplogroup D among Andaman Islanders, along with the fact that haplogroup H is descended from haplogroup CF rather than DE, suggests that the Nihali or Pulliyar are better proxies for “Ancestral South Indians” (Table S3) than the Onge^56^. “Ancestral North Indian” ancestry primarily corresponds to Southern Asian ancestry. Also, South Indian ancestry correlates best with the Dravidian language family whereas Southern Asian ancestry correlates more with the Indo-Iranian language family, consistent with a distribution throughout Persia and Pakistan^57^. Six, the Dené-Yeniseian language family, which has been proposed to show a genealogical link between Old World and New World language families^58^, is not supported by the genomic data, because the Yeniseian language correlates with Northern Asian ancestry whereas the Na-Dené language correlates with Amerindian and Circumpolar ancestries. Seven, our results are consistent with phylolinguistic hypotheses that group Altaic, Yukaghir, and Uralic (Samoyedic) languages^59, 60^, as all three correlate with Northern Asian ancestry. Since Northern Asian and Circumpolar ancestries share a common ancestor, our results also support, albeit more distantly, phylolinguistic hypotheses that group Uralic and Yukaghir languages with Eskimo-Aleut and Chukotko-Kamchatkan languages^61, 62^. Eight, the correlation of Western Asian ancestry with Northeast Caucasian, Northwest Caucasian, and Kartvelian languages is consistent with the phylolinguistic hypothesis that these three groups of languages are related in a larger grouping called Ibero-Caucasian^63^.

Our study has some limitations. Merging genetic data from different sources and platforms can be problematic; this concern is partially mitigated by the fact the markers common to all platforms tend to perform well. Also, the 1000 Genomes Project offers limited coverage of all populations and ancestries from a global perspective; hence, our comparison based on *F*_*ST*_ to address marker ascertainment bias should be viewed as encouraging but not as a final answer. Despite the large numbers of samples and individuals, our dataset is underpowered for recent events. For example, we did not detect separate ancestries corresponding to West-Central Africans, Eastern Bantu speakers, and Southern Bantu speakers resulting from the Bantu expansion^64^. Also, the absence of ancestral genotypes limits our ability to draw inferences, particularly regarding dating. A linguistic complication involves the presence of bi- or multi-lingual populations.

In summary, we found that genetic differentiation of human ancestries largely occurred subsequent to the Out-of-Africa migrations. The vast majority of present-day humans have mixed ancestry. Having estimated phylogenetic relationships among ancestries allowing for ancient gene flow, instances of mixed ancestries in which the ancestries do not share an immediate common ancestor support admixture rather than sharing of incompletely differentiated ancestries. Furthermore, the group labels continent, sample, race, and ethnicity are all imperfect descriptors of ancestry, such that ancestry is the preferred genomic classifier. We also find moderate to strong correlations between ancestries and languages at the family or branch levels, such that ancestry data support or refute several proposed linguistic relationships and linguistic data point to possible resolutions of heterogeneity in the ancestry data. Thus, ancestry data yield insight into a deeper past than linguistic data can, while linguistic data provide clarity to ancestry data.

## Materials and Methods

### Data collection and quality control

In our study, statistical populations are defined as ethno-linguistic groups. Statistical samples represent subsets of individuals from the ethno-linguistic groups. The global data set comprised 5,966 unrelated individuals, including 849 individuals from the Human Genome Diversity Project^10^, 268 individuals from the Singapore Genome Variation Project^25^, 105 individuals from west and central Africa^17^, 242 Native Americans and individuals from the Arctic and north Asia^22–24^, 453 individuals from a study of the Jewish Diaspora^16^, 16 Arabs from Qatar^20^, 95 Maasai from the International HapMap Project^15^, 176 individuals from India^18, 21^, 316 individuals from south Africa^19, 28, 29^, 200 individuals from The Caucasus^30^, 145 individuals from north Africa and the Basque Country^26^, 201 individuals from east Africa^27^, 75 individuals from Lebanon^35^, 51 individuals from Spain^32^, 258 individuals from northeast Eurasia^34, 36^, 24 individuals from the Afghan Hindu Kush^33^, and 2,492 individuals from the 1000 Genomes Project^31^. We accomplished all data management and quality control using PLINK version 1.9^65^. We generated graphics with R^66^. Maps were drawn using the R libraries maps and plotrix.

We excluded 1) all individuals or markers with genotyping call rates <95%, and 2) individuals identified as identical samples, 1st degree relatives, or 2nd degree relatives. Our resulting data set consisted of 19,075 diallelic, autosomal SNPs with experimentally determined genotypes; we did not impute missing genotypes. The genotyping call rate was 99.8%. The average distance between markers was 137.5 kb. Data are available at http://crggh.nih.gov/resources.cfm.

### SNP Ascertainment Bias

To investigate possible SNP ascertainment bias, we used the –weir-fst-pop function in VCFtools, version 0.1.14^67^. We compared pairwise *F*_*ST*_ estimates based on our panel of SNPs to pairwise estimates based on whole genome sequences^31^.

### Admixture analysis

We performed unsupervised clustering in triplicate using ADMIXTURE^68^, setting the number of ancestral components (*K*) from 1 to 40 with five-fold cross-validation. We estimated the posterior mean of *K* as the value with the minimum cross-validation error averaged over the triplicates. To confirm the posterior mean, we then estimated the posterior mode. To estimate the posterior mode, we additionally performed unsupervised clustering 30 times with *K* ranging from 1 to 30 with five-fold cross-validation. For each ancestral component, the sample with the highest percentage of that ancestral component was designated the exemplar (Table S2). To determine standard errors for the proportions of ancestral components for each individual, we repeated the ADMIXTURE analysis with the addition of 200 bootstrap replicates conditioned on *K* = 21. Accounting for both within and between individual variances, we calculated the proportions for average ancestry using inverse variance weights. We then calculated 95% confidence intervals for each ancestry and individual, zeroed out any average proportions for which the 95% confidence intervals included 0, and renormalized the remaining averages to sum to 1 (Table S3). We use these renormalized data to determine the number of ancestries present in an individual. If more than one ancestry was present in an individual, then we counted that individual as having mixed ancestry. Pairwise *F*_*ST*_ estimates between ancestral components as reported by ADMIXTURE are given in Table S4. Bounds for the significance of the correlation coefficient between *F*_*ST*_ matrices were established via a *χ* ^2^ test with one degree of freedom, given a sample size of either *N* = 18 or $\frac{N(N-1)}{2}=153$. We did not decompose *F*_*ST*_ estimates into divergence time estimates because we had insufficient data to estimate ancestry-specific effective population sizes. The ancestry-specific allele frequencies for all markers are available at http://crggh.nih.gov/resources.cfm.

When interpreting ADMIXTURE results, it is important to recognize that mixed ancestry in an individual can result from at least three different sources. (1) Admixture is defined as interbreeding between previously isolated populations^52^, in which previously isolated implies ancestrally different. (2) Shared ancestry refers to the coinheritance of more than one ancestry from the same parental source. Shared ancestry results from incomplete differentiation and is analogous to incomplete lineage sorting. (3) A non-genetic mechanism for generating mixed ancestry is assimilation.

### Power analysis

If the number of markers exceeds the number of individuals, then there exists a threshold of *F*_*ST*_ above which population structure is always detectable and that is strongly constrained by the number of individuals and weakly affected by the number of markers^69, 70^. We obtained estimates of the effective sample size per ancestry from ADMIXTURE’s Q matrix. Using the two smallest effective sample sizes and the number of markers, our studied is well powered to detect *F*_*ST*_ ≥ 0.0017. Assuming an effective population size of *N*_*e*_ = 20,000, this *F*_*ST*_ value corresponds to a divergence time *t* = 68 generations using the relationship $t=\frac{\mathrm{ln}(1-{F}_{ST})}{\mathrm{ln}(1-\frac{1}{2{N}_{e}})}$ ^71^. Assuming a generation time of 25 to 30 years^72, 73^, this divergence time corresponds to 1,700 to 2,000 years. Smaller values of *N*_*e*_ lead to smaller divergence times, meaning more recently in the past. Thus, our study is well powered for events from the origin of anatomically modern humans through the Neolithic Revolution.

### Migration analysis

We converted the output from ADMIXTURE for use with the TreeMix software^37^. First, for each ancestry, we summed the ancestry proportions across all individuals and multiplied by 2 to estimate the total allele counts. Then, for each marker, we multiplied the ancestry-specific total allele count by the ancestry-specific allele frequency. Finally, we rounded allele counts to the nearest integer. We ran TreeMix with the number of migration events set from 0 to 6, rooted with Southern African ancestry. For each number of migration events, we ran 100 random input orders. TreeMix evaluates a composite likelihood, rather than a maximum likelihood; consequently, we cannot test results using likelihood ratios. Our stopping rule was the largest number of migration events before we started to obtain positive log-likelihoods. The majority-rule consensus tree was based on 100 bootstrap replicates.

### Language analysis

We annotated each sample with language as reported in the source publications and supplemented with the Ethnologue^40^. Based on the language annotation (Table S1), we defined a binary indicator variable equaling 1 if the sample was annotated by the family or branch being tested, or 0 otherwise. For each specific ancestry-language hypothesis, we estimated the point-biserial correlation coefficient, which is mathematically equivalent to the Pearson product-moment correlation coefficient, between the proportion of that ancestry (*i*.*e*., the appropriate column of Table S3) and the binary indicator variable for language, across all samples. We then tested the correlation coefficients for significance using a *t*-test. All *p*-values reported in Table S6 are uncorrected for multiple comparisons.

### Ethics

This project was determined to be excluded from IRB Review by the National Institutes of Health Office of Human Subjects Research Protections, Protocol #12183.

## Electronic supplementary material

Supplement
