# Supplementary material for: Human ancestry correlates with language and reveals that race is not an objective genomic classifier
Source: Sci Rep. 2017 May 8;7:1572. doi: 10.1038/s41598-017-01837-7 (PMC5431528; doi:10.1038/s41598-017-01837-7)
Supplement: Supplementary file 1 — Supplement [file 41598_2017_1837_MOESM1_ESM.pdf]

Human ancestry correlates with language and reveals that race is not an objective genomic classifier

Jennifer L. Baker, Charles N. Rotimi, Daniel Shriner

Supplementary Figures S1–S2

Supplementary Tables S1–S6

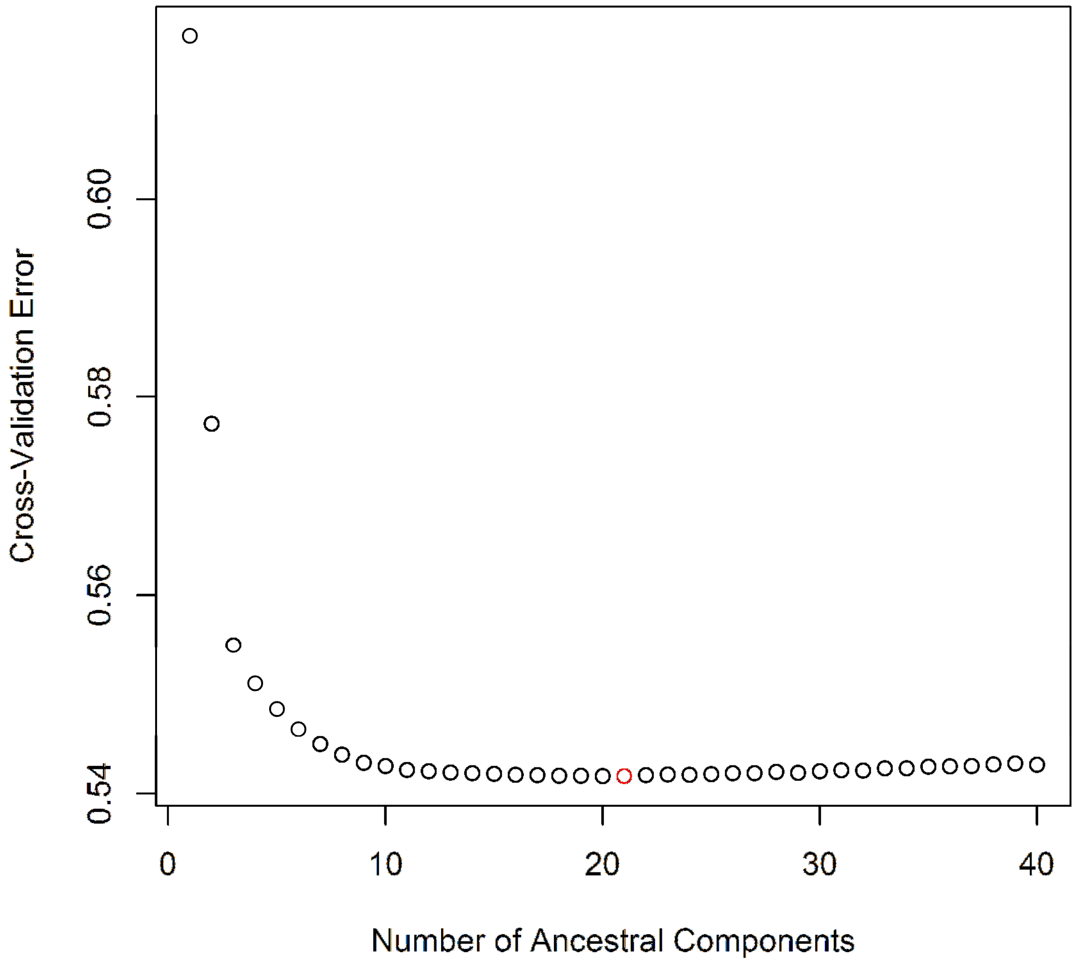

Figure S1. Cross-validation error as a function of the number of ancestral components K. The red symbol indicates the minimum cross-validation error, which occurs at $K = 21$.

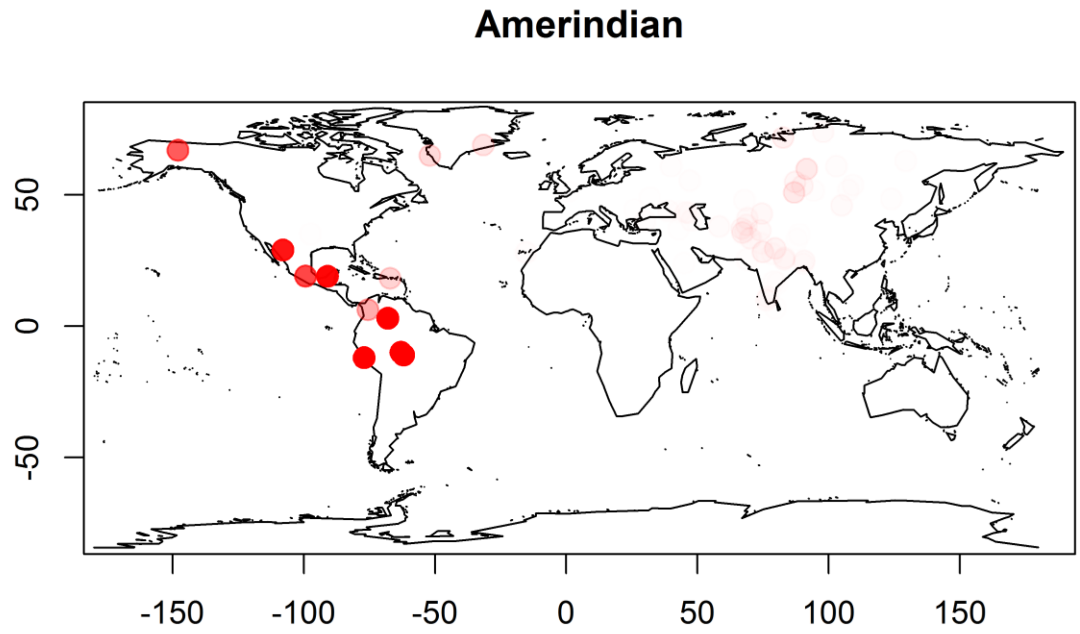

Figure S2. (A) Geographic distribution of Amerindian ancestry. The intensity of red is directly proportional to the denoised and renormalized sample mean. The map was drawn using the R (version 3.3.2, https://www.r-project.org) libraries maps and plotrix.

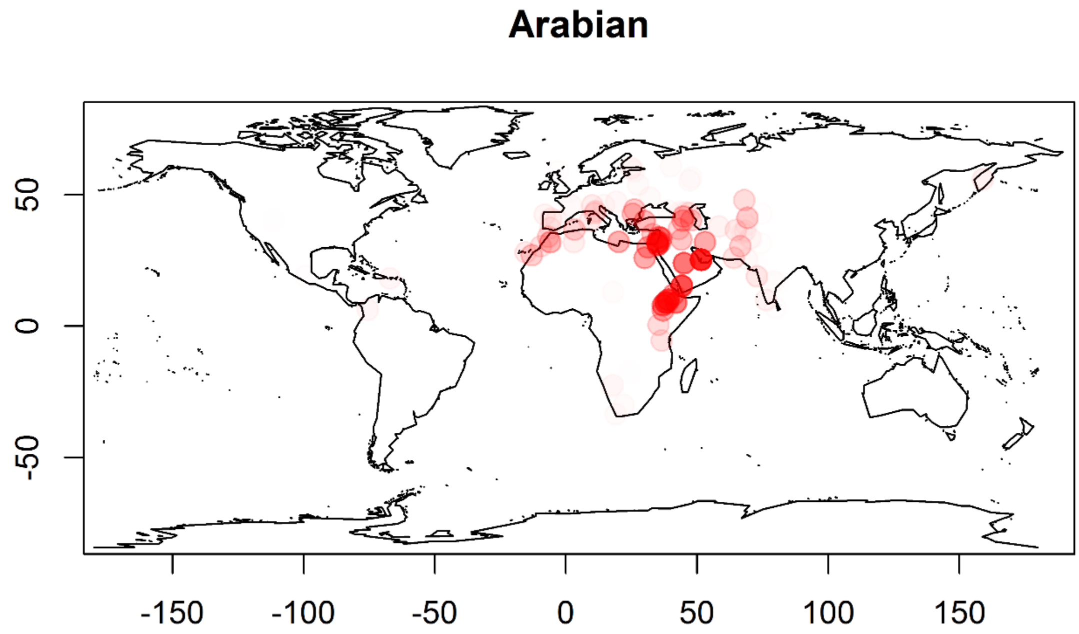

Figure S2. (B) Geographic distribution of Arabian ancestry. The intensity of red is directly proportional to the denoised and renormalized sample mean. The map was drawn using the R (version 3.3.2, https://www.r-project.org) libraries maps and plotrix.

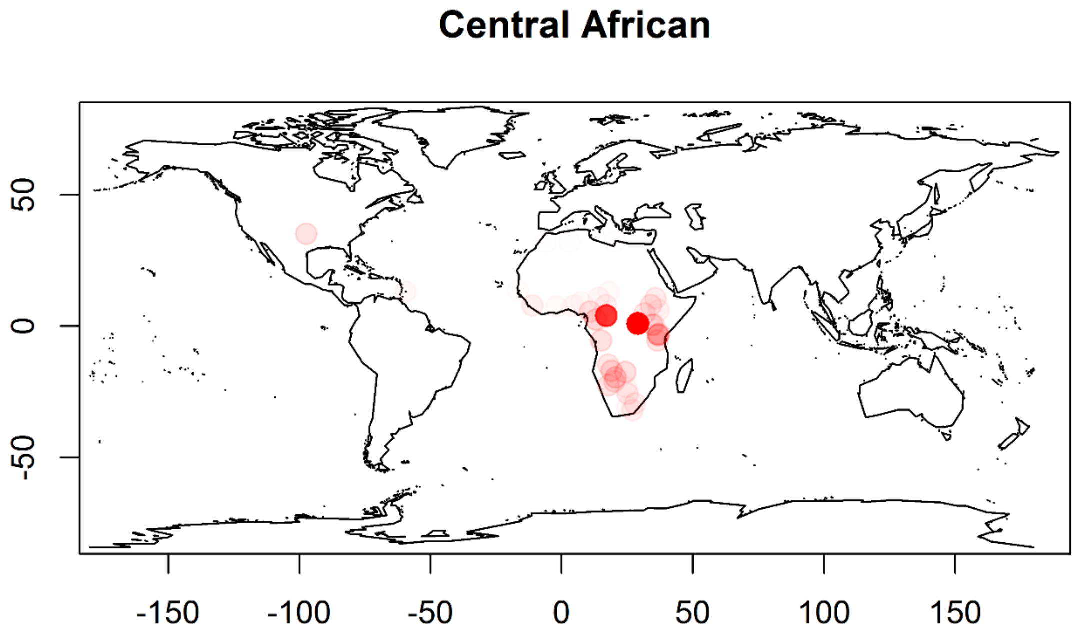

Figure S2. (C) Geographic distribution of Central African ancestry. The intensity of red is directly proportional to the denoised and renormalized sample mean. The map was drawn using the R (version 3.3.2, https://www.r-project.org) libraries maps and plotrix.

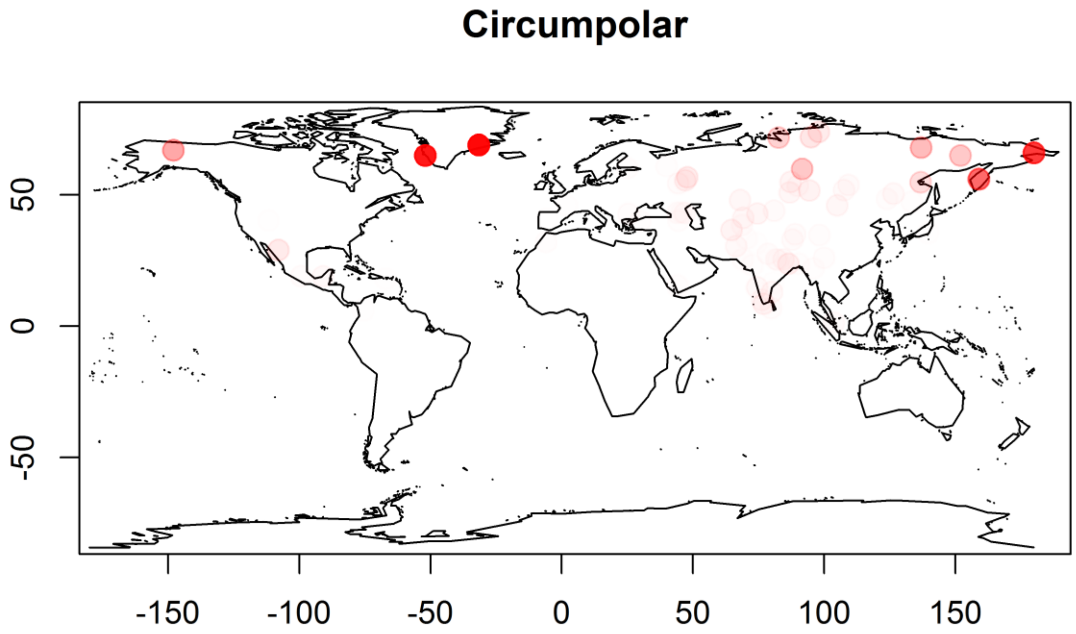

Figure S2. (D) Geographic distribution of Circumpolar ancestry. The intensity of red is directly proportional to the denoised and renormalized sample mean. The map was drawn using the R (version 3.3.2, https://www.r-project.org) libraries maps and plotrix.

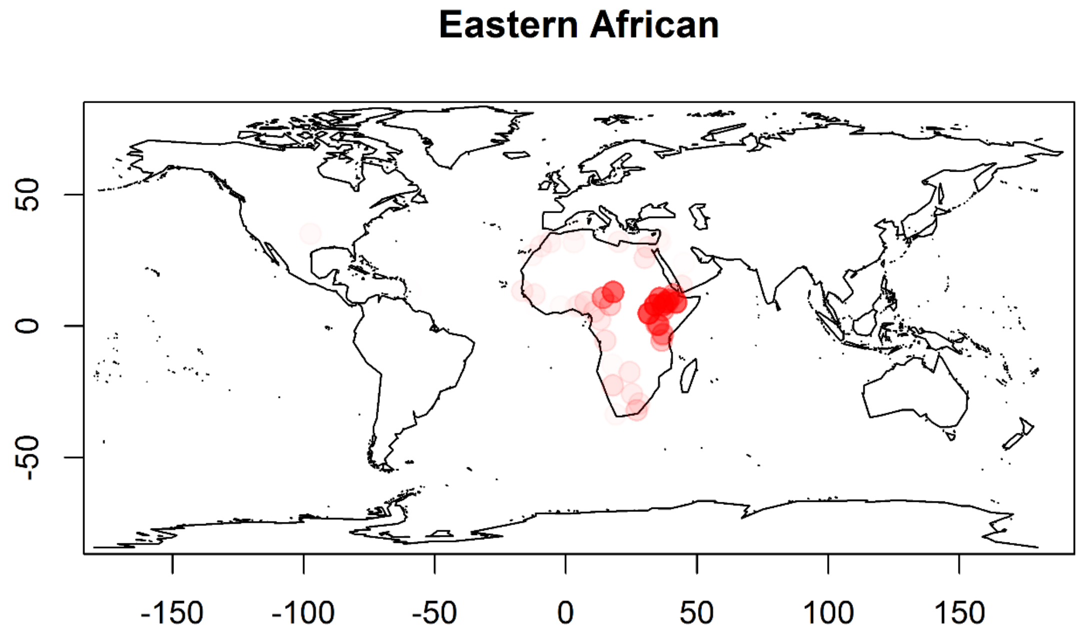

Figure S2. (E) Geographic distribution of Eastern African ancestry. The intensity of red is directly proportional to the denoised and renormalized sample mean. The map was drawn using the R (version 3.3.2, https://www.r-project.org) libraries maps and plotrix.

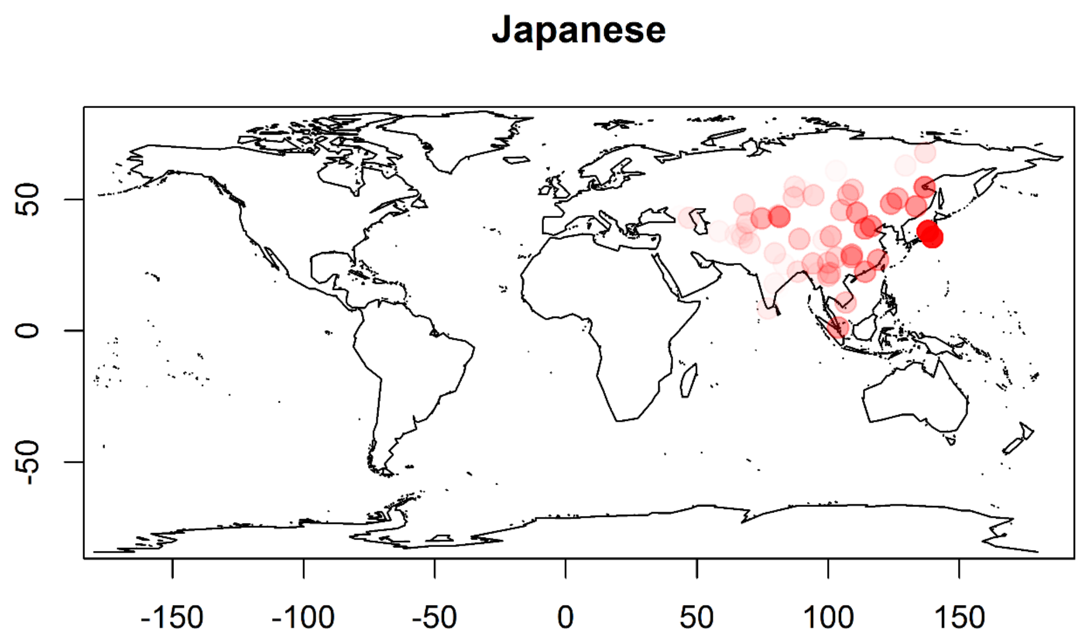

Figure S2. (F) Geographic distribution of Japanese ancestry. The intensity of red is directly proportional to the denoised and renormalized sample mean. The map was drawn using the R (version 3.3.2, https://www.r-project.org) libraries maps and plotrix.

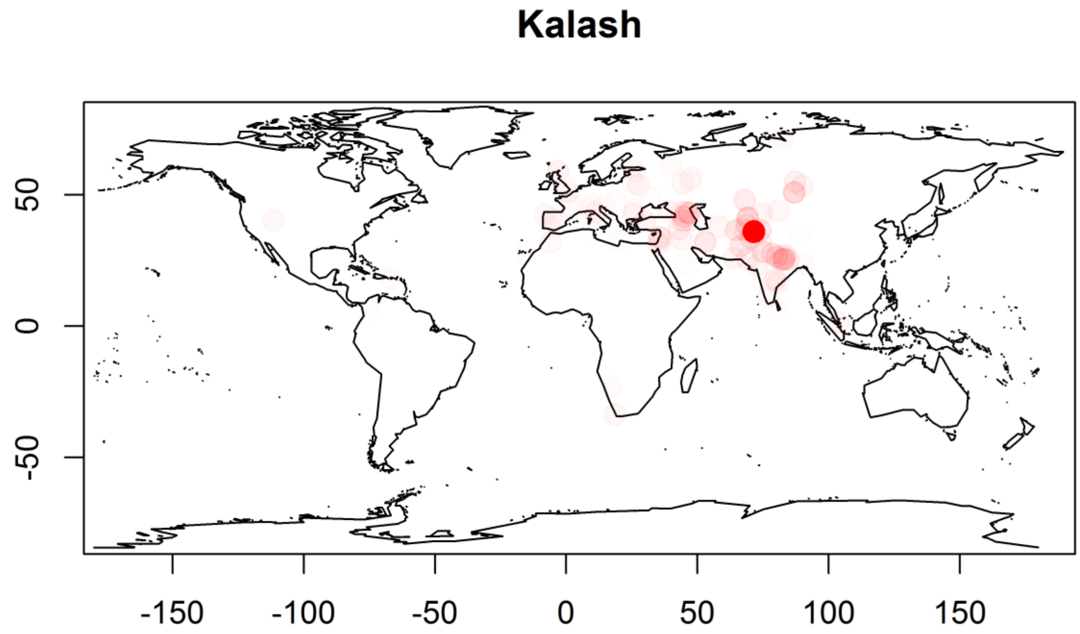

Figure S2. (G) Geographic distribution of Kalash ancestry. The intensity of red is directly proportional to the denoised and renormalized sample mean. The map was drawn using the R (version 3.3.2, https://www.r-project.org) libraries maps and plotrix.

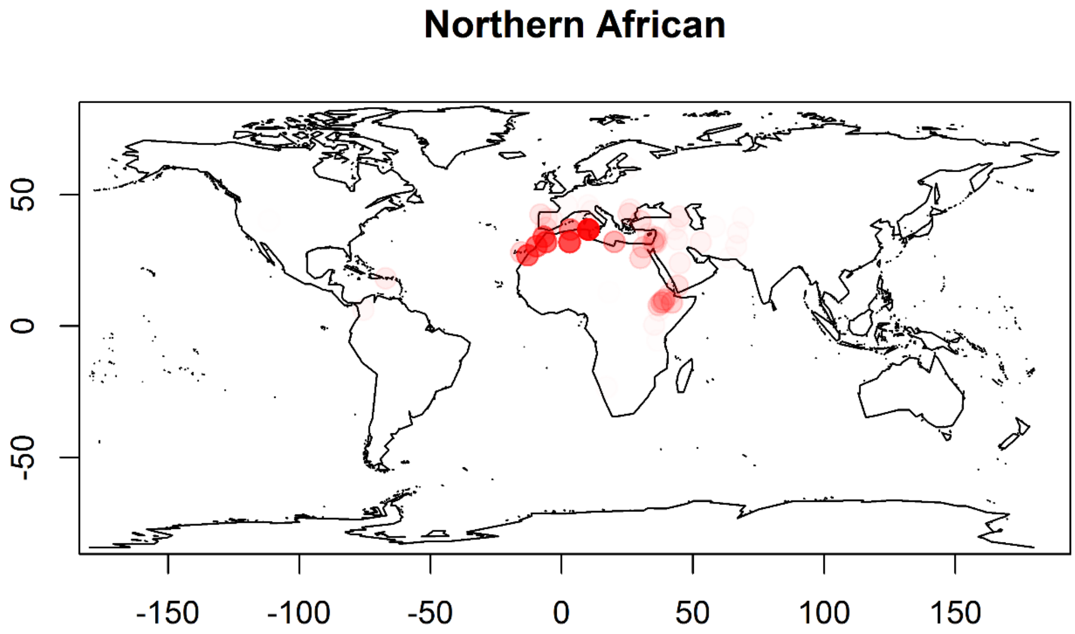

Figure S2. (H) Geographic distribution of Northern African ancestry. The intensity of red is directly proportional to the denoised and renormalized sample mean. The map was drawn using the R (version 3.3.2, https://www.r-project.org) libraries maps and plotrix.

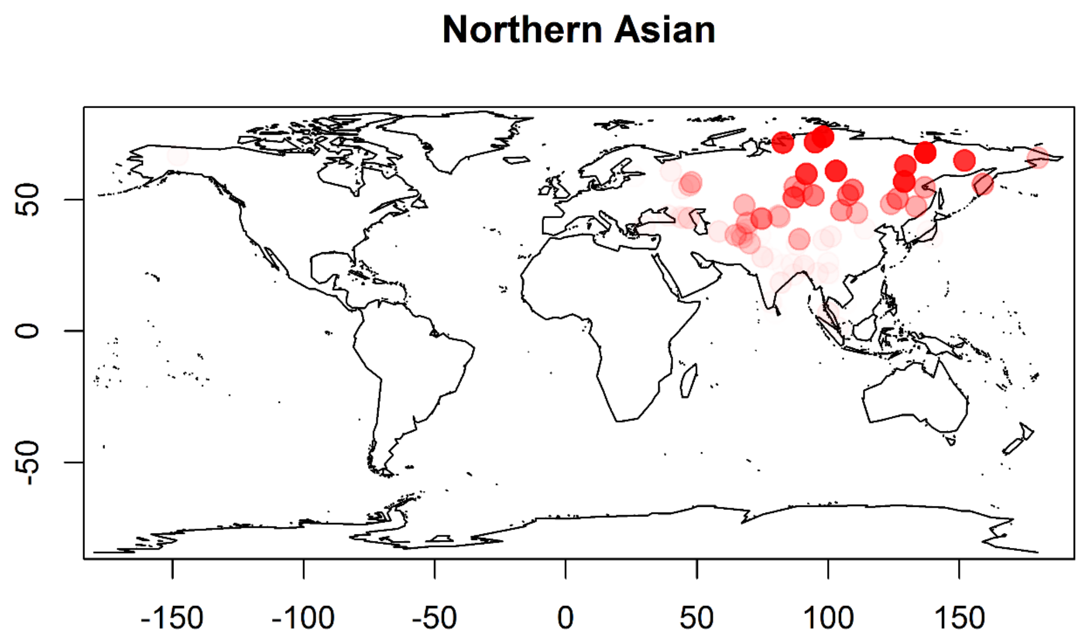

Figure S2. (I) Geographic distribution of Northern Asian ancestry. The intensity of red is directly proportional to the denoised and renormalized sample mean. The map was drawn using the R (version 3.3.2, https://www.r-project.org) libraries maps and plotrix.

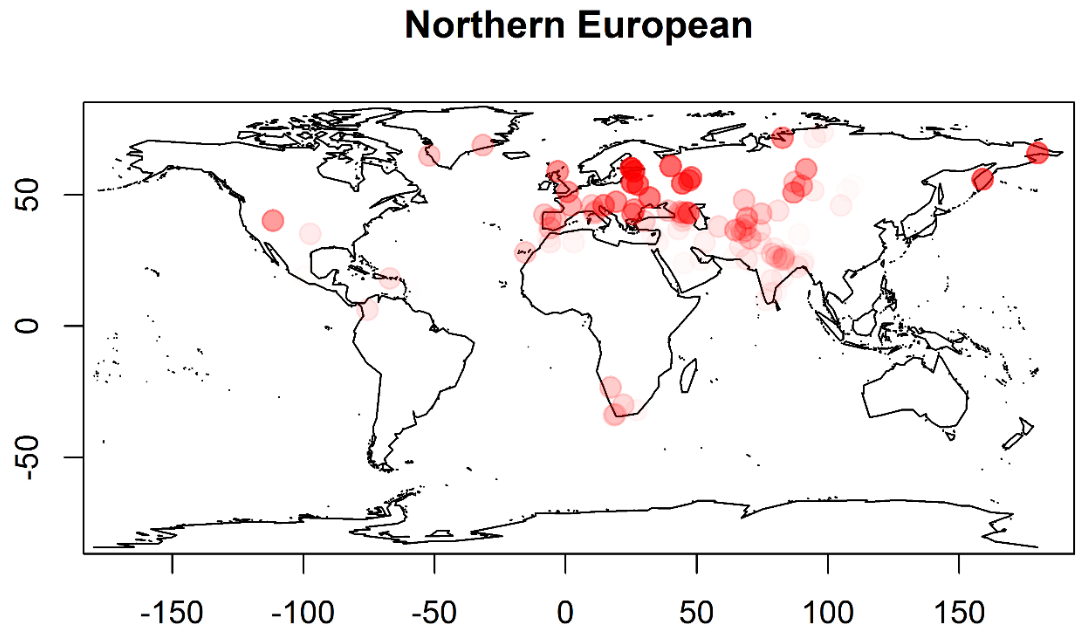

Figure S2. (J) Geographic distribution of Northern European ancestry. The intensity of red is directly proportional to the denoised and renormalized sample mean. The map was drawn using the R (version 3.3.2, https://www.r-project.org) libraries maps and plotrix.

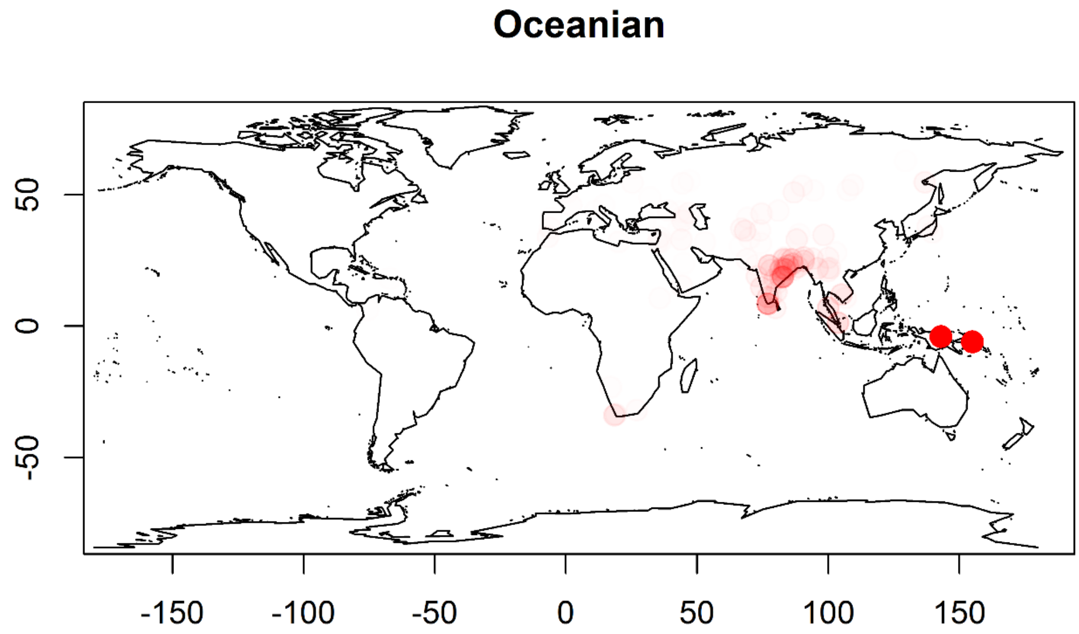

Figure S2. (K) Geographic distribution of Oceanian ancestry. The intensity of red is directly proportional to the denoised and renormalized sample mean. The map was drawn using the R (version 3.3.2, https://www.r-project.org) libraries maps and plotrix.

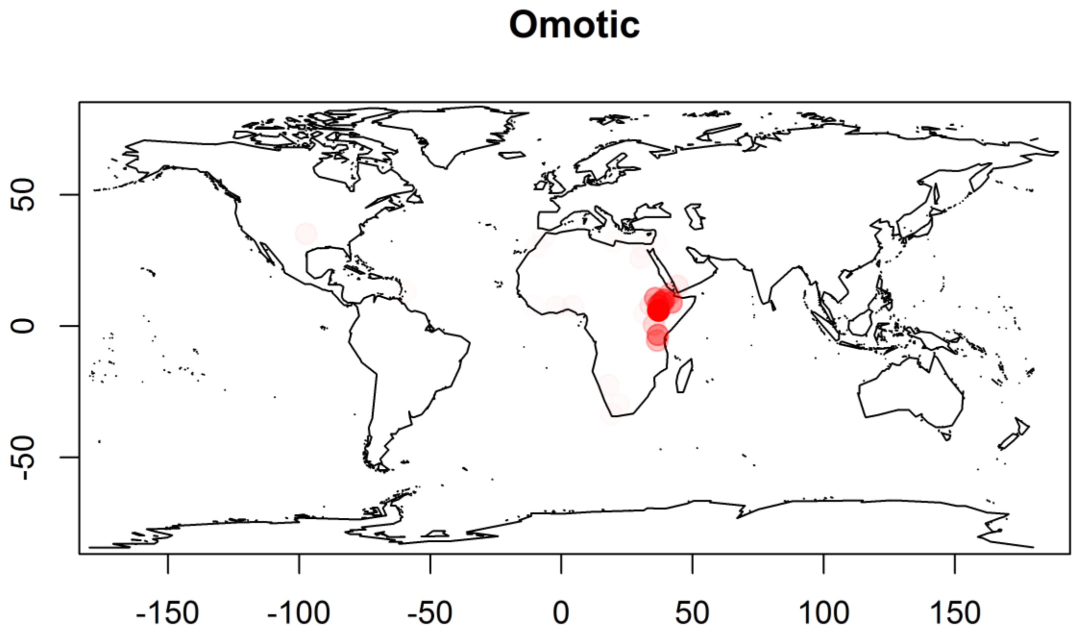

Figure S2. (L) Geographic distribution of Omotic ancestry. The intensity of red is directly proportional to the denoised and renormalized sample mean. The map was drawn using the R (version 3.3.2, https://www.r-project.org) libraries maps and plotrix.

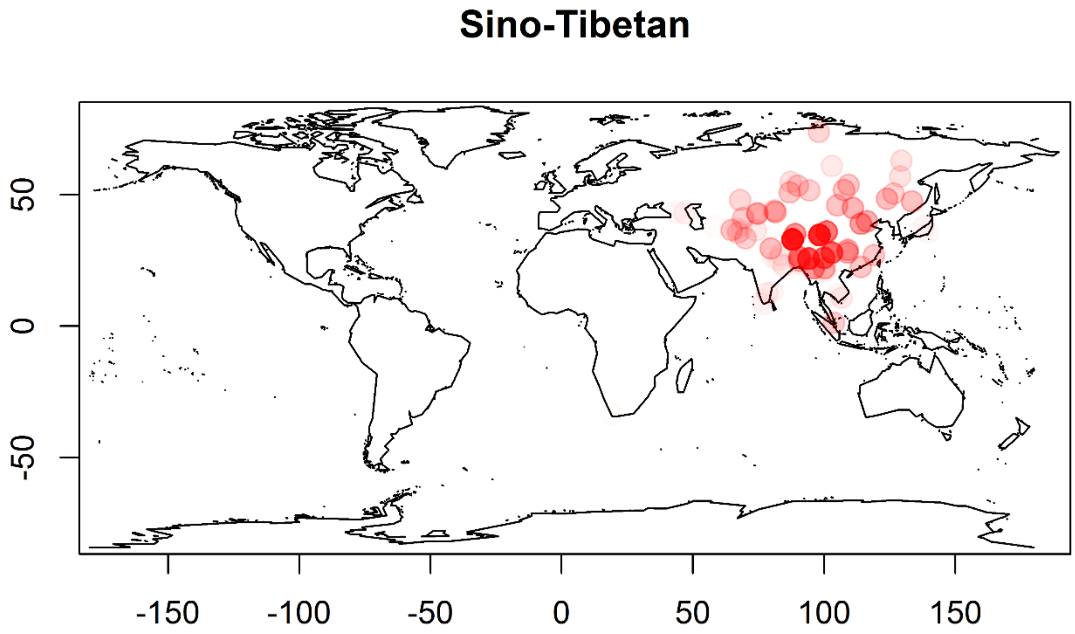

Figure S2. (M) Geographic distribution of Sino-Tibetan ancestry. The intensity of red is directly proportional to the denoised and renormalized sample mean. The map was drawn using the R (version 3.3.2, https://www.r-project.org) libraries maps and plotrix.

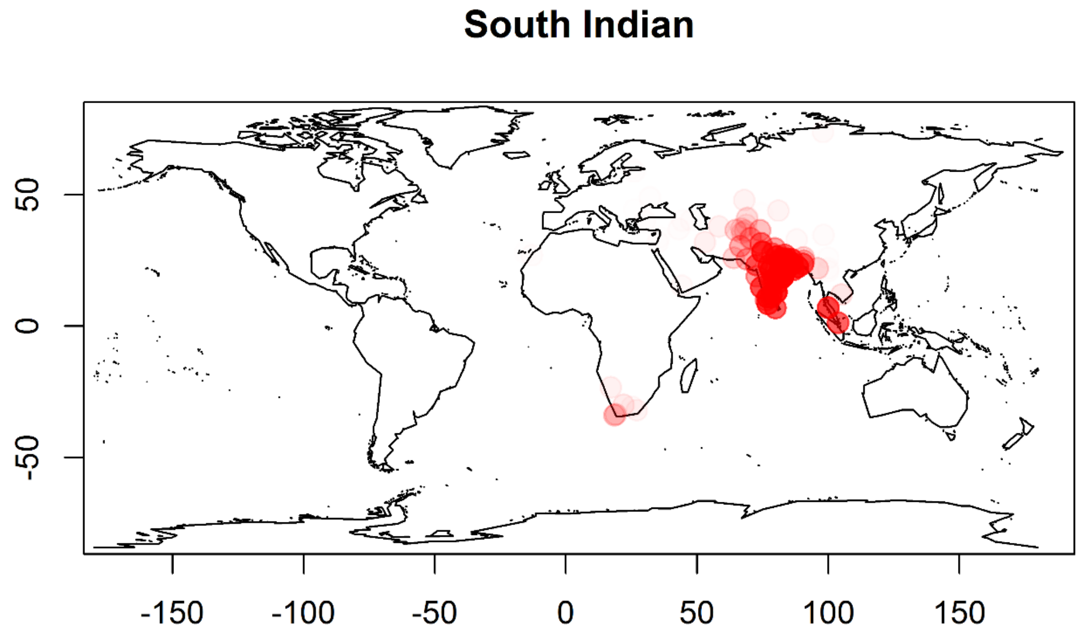

Figure S2. (N) Geographic distribution of South Indian ancestry. The intensity of red is directly proportional to the denoised and renormalized sample mean. The map was drawn using the R (version 3.3.2, https://www.r-project.org) libraries maps and plotrix.

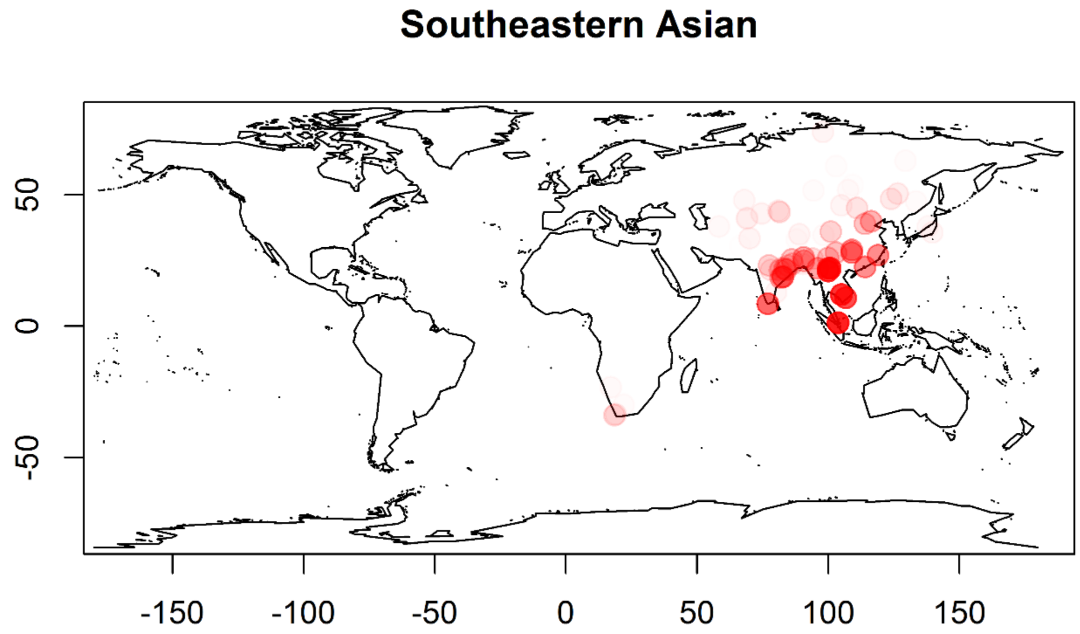

Figure S2. (O) Geographic distribution of Southeastern Asian ancestry. The intensity of red is directly proportional to the denoised and renormalized sample mean. The map was drawn using the R (version 3.3.2, https://www.r-project.org) libraries maps and plotrix.

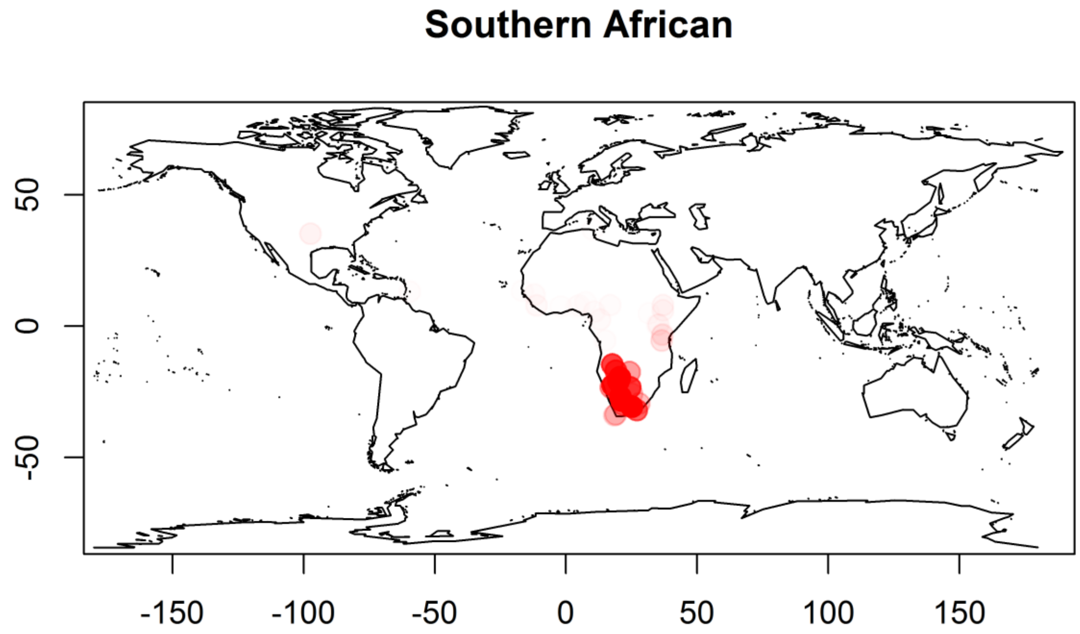

Figure S2. (P) Geographic distribution of Southern African ancestry. The intensity of red is directly proportional to the denoised and renormalized sample mean. The map was drawn using the R (version 3.3.2, https://www.r-project.org) libraries maps and plotrix.

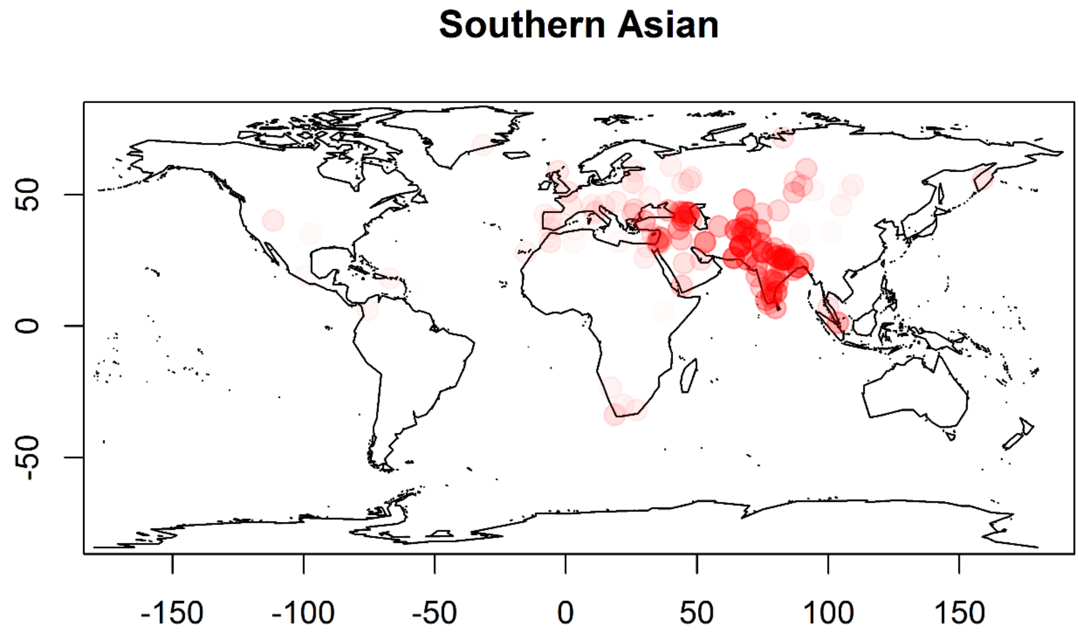

Figure S2. (Q) Geographic distribution of Southern Asian ancestry. The intensity of red is directly proportional to the denoised and renormalized sample mean. The map was drawn using the R (version 3.3.2, https://www.r-project.org) libraries maps and plotrix.

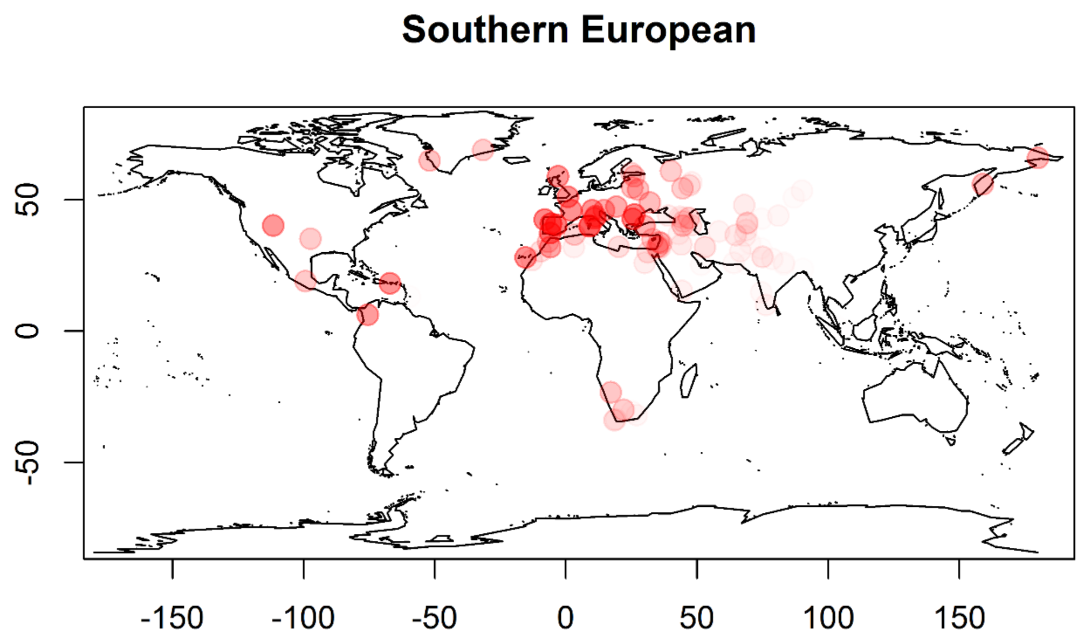

Figure S2. (R) Geographic distribution of Southern European ancestry. The intensity of red is directly proportional to the denoised and renormalized sample mean. The map was drawn using the R (version 3.3.2, https://www.r-project.org) libraries maps and plotrix.

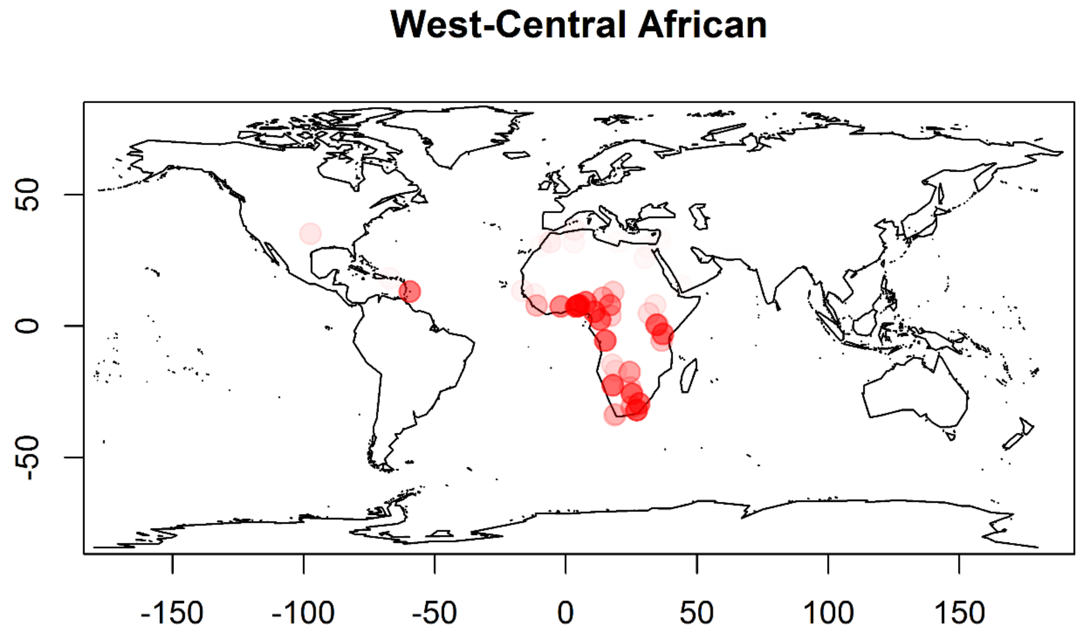

Figure S2. (S) Geographic distribution of West-Central African ancestry. The intensity of red is directly proportional to the denoised and renormalized sample mean. The map was drawn using the R (version 3.3.2, https://www.r-project.org) libraries maps and plotrix.

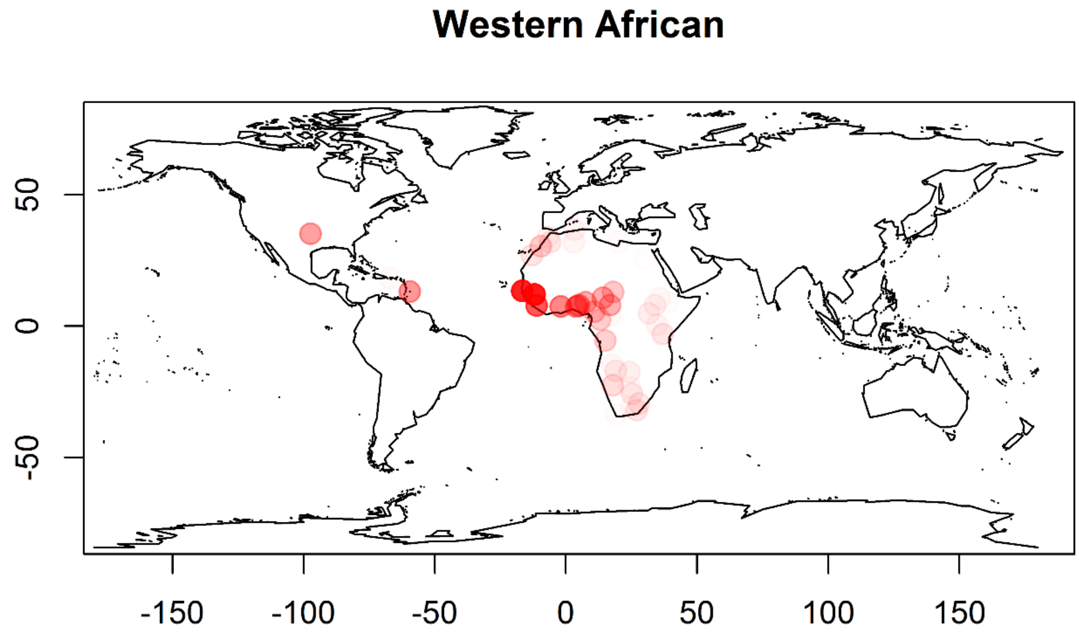

Figure S2. (T) Geographic distribution of Western African ancestry. The intensity of red is directly proportional to the denoised and renormalized sample mean. The map was drawn using the R (version 3.3.2, https://www.r-project.org) libraries maps and plotrix.

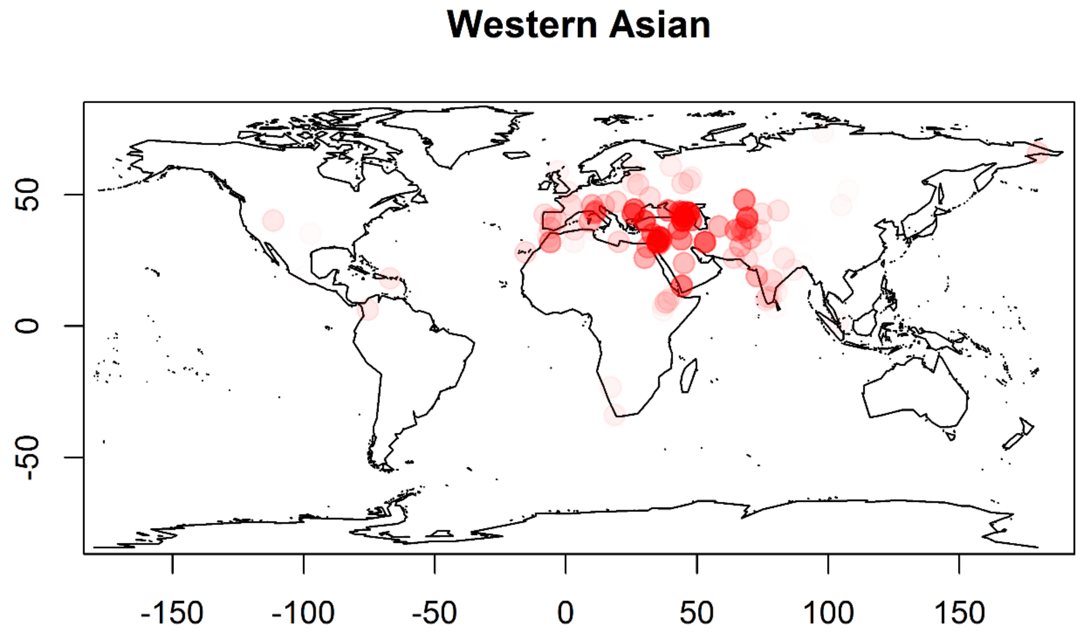

Figure S2. (U) Geographic distribution of Western Asian ancestry. The intensity of red is directly proportional to the denoised and renormalized sample mean. The map was drawn using the R (version 3.3.2, https://www.r-project.org) libraries maps and plotrix.

Table S1. Provenance, language, and reference information

Sample	Sample Size	Region	Latitude[1]	Longitude[2]	Language	Language Branch	Language Family	Reference
!Xun	17	Africa	-14.6667	17.6667	NA	Kx'a	Khoisan	29
!Xun2	8	Africa	-14.6667	17.6667	NA	Kx'a	Khoisan	28
/Gui and //Gana	9	Africa	-23.3408495	24.5019889	NA	Kwadi-Khoe	Khoisan	29
≠Khomani	32	Africa	-26.98333333	20.78333333	Khomani	Tuu	Khoisan	29
≠Khomani2	21	Africa	-28.4	21.26666667	Khomani	Tuu	Khoisan	19
Agaw	10	Africa	12	41	Agaw	Cushitic	Afroasiatic	27
Algeria	19	Africa	36.76666667	3.216666667	Berber	Berber	Afroasiatic	26
amaXhosa	15	Africa	-32	27	isiXhosa	Bantu	Niger-Congo	28
Amhara	24	Africa	10	39	Amharic	Semitic	Afroasiatic	27
Amhara	7	Africa	10	39	Amharic	Semitic	Afroasiatic	16
Angolan !Xun	4	Africa	-17	19	NA	Kx'a	Khoisan	28
Anuak	22	Africa	8	34	Anuak	Eastern Sudanic	Nilo-Saharan	27
Ari Blacksmith	11	Africa	6	37	South Omotic	Omotic	Afroasiatic	27
Ari Cultivator	21	Africa	6	37	South Omotic	Omotic	Afroasiatic	27
Bamoun	18	Africa	5.5	10.8	Bamum	Bantu	Niger-Congo	17
Bantu Kenya	10	Africa	-3	37	NA	Bantu	Niger-Congo	10
Bantu South Africa	6	Africa	-25.8	25.2	NA	Bantu	Niger-Congo	10
Basters	30	Africa	-23.31666667	17.08333333	Afrikaans	Germanic	Indo-European	28
Biaka Pygmy	21	Africa	4	17	NA	NA	NA	10
Brong	8	Africa	7.5	-2	Akan	non-Bantu	Niger-Congo	17
Bulala	15	Africa	13	18	Naba	Central Sudanic	Nilo-Saharan	17
Coloured Colesberg	18	Africa	-30.71666667	25.1	NA	NA	NA	29
Coloured District 6	8	Africa	-33.93277778	18.43416667	NA	NA	NA	28
Coloured Eastern Cape	7	Africa	-32	27	NA	NA	NA	28
Coloured Northern Cape	10	Africa	-30	22	NA	NA	NA	28
Coloured Wellington	17	Africa	-33.63333333	18.98333333	NA	NA	NA	29
Egypt	19	Africa	30.03333333	31.21666667	NA	NA	NA	26
Egypt2	12	Africa	26	30	NA	NA	NA	16
ESN	99	Africa	7.738888889	4.436111111	Esan	non-Bantu	Niger-Congo	31
Ethiopia Jew	12	Africa	9.03	38.74	NA	NA	NA	16
Ethiopian Somali	8	Africa	9	42	Somali	Cushitic	Afroasiatic	27
Fang	15	Africa	2.5	13	NA	Bantu	Niger-Congo	17
Gumuz	16	Africa	10.63888889	35.73305556	Gumuz	NA	Nilo-Saharan	27
GWD	113	Africa	13.43333333	-16.66666667	Mandinka	Mande	Niger-Congo	31
Hadza	12	Africa	-3.333333333	36.75	NA	NA	Hadza	19
Hausa	12	Africa	9.1	7.5	NA	Chadic	Afroasiatic	17
Ju/'hoan	3	Africa	-19.5931	20.5022	Ju/'hoan	Kx'a	Khoisan	28
Ju/'hoansi	2	Africa	-19.5931	20.5022	Ju/'hoan	Kx'a	Khoisan	29
Kaba	16	Africa	8	16.8	Kaba	Central Sudanic	Nilo-Saharan	17
Karretjie	16	Africa	-30.71666667	25.1	NA	Tuu	Khoisan	29
Khwe	16	Africa	-17.50388889	24.275	Khwe	Kwadi-Khoe	Khoisan	29
Kongo	9	Africa	-5.5	15	Kongo	Bantu	Niger-Congo	17
Libya	17	Africa	32.11666667	20.06666667	NA	NA	NA	26
LWK	98	Africa	0.5	34.58333333	Luhya	Bantu	Niger-Congo	31
Mada	12	Africa	10.8	14.1	NA	Chadic	Afroasiatic	17
Mandenka	21	Africa	12	-12	Mandinka	Mande	Niger-Congo	10
Mbuti Pygmy	10	Africa	1	29	NA	NA	NA	10
MKK	95	Africa	0.516666667	35.28333333	Maasai	Eastern Sudanic	Nilo-Saharan	15
Morocco	10	Africa	32	-6	Berber	Berber	Afroasiatic	16
Morocco Jew	15	Africa	32	-6	NA	NA	NA	16
Mozabite	28	Africa	32	3	Mozabite	Berber	Afroasiatic	10
MSL	85	Africa	7.875833333	-11.19083333	Mende	Mande	Niger-Congo	31
Nama	19	Africa	-22.57	17.85	Khoekhoe	Kwadi-Khoe	Khoisan	29
North Morocco	18	Africa	34.020882	-6.84165	Berber	Berber	Afroasiatic	26
Oromo	7	Africa	8	37	Oromo	Cushitic	Afroasiatic	16
Oromo	21	Africa	8	37	Oromo	Cushitic	Afroasiatic	27
Sahrawi	18	Africa	27	-13	Berber	Berber	Afroasiatic	26
San	2	Africa	-21	20	NA	Kx'a	Khoisan	10
Sandawe	23	Africa	-5.5	36.5	NA	NA	Sandawe	19
Somali	22	Africa	9	42	Somali	Cushitic	Afroasiatic	27
South Morocco	16	Africa	30.43333333	-9.6	Berber	Berber	Afroasiatic	26
Southeast Bantu	18	Africa	-29.46666667	27.93333333	NA	Bantu	Niger-Congo	29
Southwest Bantu	11	Africa	-22.57	17.85	NA	Bantu	Niger-Congo	29
Sudanese	20	Africa	4.85	31.6	Dinka-Nuer	Eastern Sudanic	Nilo-Saharan	27
Tigray	5	Africa	9	38	Tigrinya	Semitic	Afroasiatic	16

Tunisia	18	Africa	36.8	10.18333333	Berber	Berber	Afroasiatic	26
Tygray	20	Africa	9	38	Tigrinya	Semitic	Afroasiatic	27
Wolayta	6	Africa	6	37	Wolayta	North Omotic	Afroasiatic	27
Yoruba	21	Africa	8	5	Yoruba	non-Bantu	Niger-Congo	10
YRI	108	Africa	7.396388889	3.916666667	Yoruba	non-Bantu	Niger-Congo	31
ACB	95	America	13.16666667	-59.55	NA	NA	NA	31
ASW	56	America	35.21666667	-97.41666667	NA	NA	NA	31
Athabask	15	America	67	-148	Athabaskan	NA	Na-Dené	24
CEU	99	America	40.24444444	-111.6608333	English	Germanic	Indo-European	31
CLM	94	America	6.235925	-75.57513611	Spanish	NA	NA	31
Colombian	2	America	3	-68	Piapoco	Arawakan	Amerind	10
East Greenland	7	America	69	-31.6	Inuit	Eskimo	Eskimo-Aleut	24
East Greenland2	2	America	69	-31.6	Inuit	Eskimo	Eskimo-Aleut	22
Karitiana	2	America	-10	-63	Karitiâna	Tupian	Amerind	10
Maya	18	America	19	-91	Mayan	Mayan	Amerind	10
MXL	64	America	19.05	-99.36666667	NA	NA	NA	31
PEL	85	America	-12.04333333	-77.02833333	NA	Quechumaran	Amerind	31
Pima	2	America	29	-108	O'odham	Uto-Aztecan	Amerind	10
PUR	104	America	18.20111111	-67.13972222	NA	NA	NA	31
Surui	1	America	-11	-62	Surui	Tupian	Amerind	10
West Greenland	10	America	65	-52	Inuit	Eskimo	Eskimo-Aleut	24
West Greenland2	1	America	65	-52	Inuit	Eskimo	Eskimo-Aleut	22
Altaian	13	Asia	50.91666667	86.91666667	Altay	Siberian Turkic	Turkic	24
Altaian2	4	Asia	50.91666667	86.91666667	Altay	Siberian Turkic	Turkic	23
Arunthathiyar	4	Asia	13.09	80.27	NA	NA	Dravidian	16
Asur	2	Asia	23.76	86.42	Asuri	Munda	Austroasiatic	18
Balochi	24	Asia	30.5	66.5	Balochi	Indo-Iranian	Indo-European	10
BEB	86	Asia	23.7	90.36666667	Bengali	Indo-Iranian	Indo-European	31
Bengali	1	Asia	22.56	88.4	Bengali	Indo-Iranian	Indo-European	21
Bhunjia	1	Asia	21.27	81.56	Halbi	Indo-Iranian	Indo-European	21
Bonda	4	Asia	18.4	81.88	Bonda	Munda	Austroasiatic	18
Brahmins from Tamil Nadu	2	Asia	12.49	78.42	Telugu	NA	Dravidian	21
Brahmins from Uttar Pradesh	8	Asia	26.06	83.18	NA	NA	Indo-European	21
Brahui	24	Asia	30.5	66.5	Brahui	NA	Dravidian	10
Burmese	15	Asia	22	96	Burmese	NA	Sino-Tibetan	18
Burusho	23	Asia	36.5	74	NA	NA	Burushaski	10
Buryat	17	Asia	53.8	109.3333333	Buryat	NA	Mongolic	24
Buryat2	25	Asia	51.83333333	107.6	Buryat	NA	Mongolic	36
Cambodian	8	Asia	12	105	Khmer	Mon-Khmer	Austroasiatic	10
CDX	92	Asia	22	100.8	NA	Southwestern Tai	Tai-Kadai	31
Chamar	10	Asia	25.37	83.04	NA	NA	Indo-European	21
CHB	103	Asia	39.91388889	116.3916667	Chinese	NA	Sino-Tibetan	31
Chenchu	4	Asia	18	79.59	Chenchu	NA	Dravidian	21
CHS	105	Asia	22.55	114.1	Chinese	NA	Sino-Tibetan	31
Chukchi	14	Asia	66	180	Chukchi	NA	Chukotko-Kamchatkan	24
Chukchi2	1	Asia	66	180	Chukchi	NA	Chukotko-Kamchatkan	34
Dai	10	Asia	21	100	NA	Southwestern Tai	Tai-Kadai	10
Daur	9	Asia	48.5	124	Daur	NA	Mongolic	10
ongolians from Qinghai-Tibeta	42	Asia	35	89	Mongolian	NA	Mongolic	36
Dharkar	11	Asia	25.44	83.1	NA	Indo-Iranian	Indo-European	21
Dhurwa	1	Asia	18.78	82.68	Duruwa	NA	Dravidian	21
Dolgan	7	Asia	74	98	Dolgan	Siberian Turkic	Turkic	24
Dolgan2	3	Asia	74	98	Dolgan	Siberian Turkic	Turkic	34
Dusadh	6	Asia	25.44	84.56	Hindi	Indo-Iranian	Indo-European	21
Even	8	Asia	68	137	Even	NA	Tungusic	34
Even2	1	Asia	68	137	Even	NA	Tungusic	23
Evenki	14	Asia	61	103	Evenki	NA	Tungusic	24
Gadaba	1	Asia	18.79	82.7	NA	Munda	Austroasiatic	18
Garo	4	Asia	26.17	90.62	Garo	NA	Sino-Tibetan	18
GIH	102	Asia	23.21666667	72.68333333	Gujarati	Indo-Iranian	Indo-European	31
Gond	4	Asia	22.1	82.16	Gondi	NA	Dravidian	21
Hakkipikki	4	Asia	14.79	74.51	NA	NA	Dravidian	21
Han North China	10	Asia	39	114	Chinese	NA	Sino-Tibetan	10
Han South China	34	Asia	36	114	Chinese	NA	Sino-Tibetan	10
Hazara	22	Asia	33.5	70	Hazaragi	Indo-Iranian	Indo-European	10
Hazara2	5	Asia	35.61	67.21	Hazaragi	Indo-Iranian	Indo-European	33
Hezhen	8	Asia	47.5	133.5	Nanai	NA	Tungusic	10
Ho	5	Asia	25.4	86.13	Ho	Munda	Austroasiatic	18
ITU	102	Asia	16.5	80.64	Telugu	NA	Dravidian	31

Japanese	28	Asia	38	138	Japanese	NA	Japonic	10
JPT	104	Asia	35.68950556	139.6917	Japanese	NA	Japonic	31
Juang	2	Asia	21.49	83.98	Juang	Munda	Austroasiatic	18
Kalash	23	Asia	36	71.5	Kalasha	Indo-Iranian	Indo-European	10
Kanjar	5	Asia	26.45	80.32	NA	Indo-Iranian	Indo-European	21
Kanjars	2	Asia	26.45	80.32	NA	Indo-Iranian	Indo-European	21
Kazakhstan	18	Asia	48	68	Kazakh	Kipchak	Turkic	23
Ket	2	Asia	59.88333333	91.66666667	Ket	NA	Yeniseian	24
Ket2	2	Asia	59.88333333	91.66666667	Ket	NA	Yeniseian	34
Khakas	17	Asia	53.5	90	Khakas	Siberian	Turkic	34
Kharia	2	Asia	21.89	83.36	Kharia	Munda	Austroasiatic	18
Khasi	3	Asia	24.87	90.72	Khasic	Khasi-Palaungic	Austroasiatic	18
KHV	99	Asia	10.76944444	106.6819444	Vietnamese	Vietic	Austroasiatic	31
Kochi Jew	4	Asia	9.97	76.28	NA	NA	NA	16
Kol	16	Asia	25.15	82.58	NA	NA	Indo-European	21
Koryak	9	Asia	56	159	Koryak	Chukotkan	Chukotko-Kamchatkan	24
Koryak2	1	Asia	56	159	Koryak	Chukotkan	Chukotko-Kamchatkan	34
Kshatriya	7	Asia	27.56	78.65	NA	NA	Indo-European	21
Kurd	6	Asia	48	68	Kurdish	Indo-Iranian	Indo-European	30
Kurmi	1	Asia	22.56666667	88.36666667	Kurmali	Indo-Iranian	Indo-European	21
Kurumba	4	Asia	21.46	86.78	Kurumba	NA	Dravidian	21
Kyrgyzstan	18	Asia	42.86666667	74.6	Kyrgyz	Kipchak	Turkic	23
Kyrgyzstani	25	Asia	42.86666667	74.6	Kyrgyz	Kipchak	Turkic	36
Lahu	7	Asia	22	100	Lahu	NA	Sino-Tibetan	10
Lambadi	1	Asia	17.45	78.5	NA	NA	Dravidian	21
Maduo Tibetan	31	Asia	34.7	98.21666667	Tibetan	NA	Sino-Tibetan	36
Makrani	25	Asia	26	64	Makrani	Indo-Iranian	Indo-European	10
Malaya	1	Asia	7	100	NA	NA	Dravidian	16
Mawasi	1	Asia	23.15	77.42	NA	Munda	Austroasiatic	21
Meena	1	Asia	28.29	74.97	NA	Indo-Iranian	Indo-European	21
Meghawal	1	Asia	28.27	74.95	NA	NA	Indo-European	21
Miaozu	10	Asia	28	109	Hmongic	NA	Hmong-Mien	10
Mongolia	10	Asia	45	111	Mongolian	NA	Mongolic	10
Mongolia2	9	Asia	46	105	Mongolian	NA	Mongolic	24
Mumbai Jew	4	Asia	18.975	72.82583333	NA	NA	NA	16
Muslim	5	Asia	25.44	81.83	NA	NA	NA	21
Naga	4	Asia	25.67	94.11	Naga	Tibeto-Burman	Sino-Tibetan	21
Naxi	7	Asia	26	100	Naxi	NA	Sino-Tibetan	10
Nganassan	10	Asia	72	95	Nganasan	Samoyedic	Uralic	24
Nihali	2	Asia	21.38	78.15	NA	NA	Nihali	21
Nivkh	3	Asia	54.8	136.8333333	NA	NA	Nivkh	34
North Kannadi	8	Asia	8.5074	76.972	Kannada	NA	Dravidian	16
Oroqen	9	Asia	50.5	126.5	Oroqen	NA	Tungusic	10
Paniya	3	Asia	8.5074	76.972	Paniya	NA	Dravidian	16
Pashtun	5	Asia	36.29	68.29	Pashto	Indo-Iranian	Indo-European	33
Pathan	22	Asia	33.5	70.5	Pashto	Indo-Iranian	Indo-European	10
Piramalai Kallars	8	Asia	10.99	78.22	Tamil	NA	Dravidian	21
PJL	96	Asia	31.54972222	74.34361111	Punjabi	Indo-Iranian	Indo-European	31
Pulliyar	5	Asia	11.02	76.98	Malayalam	NA	Dravidian	21
Santhal	1	Asia	23.08	84.66	Santali	Munda	Austroasiatic	18
Savara	2	Asia	18.8	82.7	Sora	Munda	Austroasiatic	18
cheduled Caste from Tamil Nac	2	Asia	13.05	80.17	Tamil	NA	Dravidian	21
heduled Caste from Uttar Prade	5	Asia	25.42	83.1	NA	NA	Indo-European	21
Selkup	10	Asia	71.83333333	82.66666667	Selkup	Samoyedic	Uralic	24
Selkup2	7	Asia	71.83333333	82.66666667	Selkup	Samoyedic	Uralic	23
She	9	Asia	27	119	She	NA	Hmong-Mien	10
Shor	4	Asia	54.93333333	87.23333333	Shor	Siberian	Turkic	34
Sindhi	24	Asia	25.5	69	Sindhi	Indo-Iranian	Indo-European	10
Singapore Chinese	96	Asia	1.3	103.8	Chinese	NA	Sino-Tibetan	25
Singapore Indian	83	Asia	1.3	103.8	NA	NA	NA	25
Singapore Malay	89	Asia	1.3	103.8	NA	NA	NA	25
STU	99	Asia	6.933333333	79.86666667	Tamil	NA	Dravidian	31
Tajikistan	5	Asia	37.18	66.9	Tajik	Indo-Iranian	Indo-European	33
Tajikistan2	15	Asia	38.55	68.8	Tajik	Indo-Iranian	Indo-European	30
Tharu	2	Asia	27.12	83.45	Tharu	Indo-Iranian	Indo-European	21
Tibetans from Tuo Tuo River	46	Asia	33	88	Tibetan	NA	Sino-Tibetan	36
Tu	10	Asia	36	101	NA	NA	Monguor	10
Tujia	10	Asia	29	109	Tujia	NA	Sino-Tibetan	10
Turkmenistan	4	Asia	36.6	64.74	Turkmen	Oghuz	Turkic	33

Turkmenistan2	11	Asia	37.96666667	58.33333333	Turkmen	Oghuz	Turkic	30
Tuvinian	14	Asia	51.698	94.39	Tuvan	Siberian Turkic	Turkic	24
Uttaranchal	1	Asia	29.6	79.65	Hindi	Indo-Iranian	Indo-European	21
Uygur	10	Asia	44	81	Uyghur	NA	Turkic	10
Uzbekistan	15	Asia	41.26666667	69.21666667	Uzbek	NA	Turkic	16
Uzbekistan Jew	2	Asia	41.26666667	69.21666667	NA	NA	NA	16
Uzbekistan2	5	Asia	36.6	64.74	Uzbek	NA	Turkic	33
Uzbekistan3	4	Asia	41	69	Uzbek	NA	Turkic	23
Velama	10	Asia	17.05	79.27	Telugu	NA	Dravidian	21
Xibo	9	Asia	43.5	81.5	Xibe	NA	Tungusic	10
Yakut	25	Asia	63	129.5	Yakut	Siberian Turkic	Turkic	10
Yakut2	1	Asia	57	129	Yakut	Siberian Turkic	Turkic	34
Yizu	10	Asia	28	103	NA	NA	Sino-Tibetan	10
Yukaghir	6	Asia	65	152	NA	NA	Yukaghir	24
Abkhasian	20	Caucasus	43	40.98333333	Abkhaz	Abazgi	Northwest Caucasian	30
Adygei	15	Caucasus	44	39	Adyghe	Circassian	Northwest Caucasian	10
Armenia	19	Caucasus	40.18333333	44.51666667	NA	Armenian	Indo-European	16
Armenia2	16	Caucasus	40.18333333	44.51666667	NA	Armenian	Indo-European	30
Azerbaijan Jew	8	Caucasus	40.3	47.7	NA	NA	NA	16
Balkar	19	Caucasus	43.58333333	43.4	Karachay-Balkar	Kipchak	Turkic	30
Chechen	20	Caucasus	43.4	45.71666667	Chechen	NA	Northeast Caucasian	30
Georgia	20	Caucasus	41.71666667	44.78333333	Georgian	NA	Kartvelian	16
Georgia Jew	4	Caucasus	41.71666667	44.78333333	NA	NA	NA	16
Kumyk	14	Caucasus	43.1	46.88333333	Kumyk	Kipchak	Turkic	30
Lezgian	18	Caucasus	43.1	46.88333333	Lezgian	Lezgic	Northeast Caucasian	16
Nogai	16	Caucasus	43.1	46.88333333	Nogai	Kipchak	Turkic	30
North Ossetia	15	Caucasus	43.18333333	44.23333333	Ossetian	Indo-Iranian	Indo-European	30
Andalusia	17	Europe	37.38333333	-5.983333333	Spanish	Italic	Indo-European	32
Ashkenazi East	9	Europe	NA	NA	Yiddish	Germanic	Indo-European	16
Ashkenazi West	9	Europe	NA	NA	Yiddish	Germanic	Indo-European	16
Basque	24	Europe	43	0	NA	NA	Basque	10
Basque2	20	Europe	42.88194444	-1.916944444	NA	NA	Basque	26
Belorussia	9	Europe	53.91666667	27.55	Belarussian	Balto-Slavic	Indo-European	16
Bulgaria	13	Europe	42.75	25.5	Bulgarian	Balto-Slavic	Indo-European	30
Bulgaria Jew	9	Europe	42.75	25.5	NA	NA	NA	16
Canary Islands	17	Europe	28.1	-15.4	Spanish	NA	NA	32
Chuvash	17	Europe	55.55	47.1	Chuvash	Oghur	Turkic	16
Estonia	15	Europe	59	26	Estonian	Finnic	Uralic	23
FIN	99	Europe	60.17083	24.9375	Finnish	Finnic	Uralic	31
French	28	Europe	46	2	French	Italic	Indo-European	10
Galicia	17	Europe	42.5	-8.1	NA	Italic	Indo-European	32
GBR	91	Europe	51.19	0.73	English	Germanic	Indo-European	31
Hungary	19	Europe	47.43333333	19.25	Hungarian	Finno-Ugric	Uralic	16
IBS	107	Europe	40.961612	-5.667607	Spanish	Italic	Indo-European	31
Lithuania	10	Europe	54.68333333	25.31666667	Lithuanian	Balto-Slavic	Indo-European	16
Mari	15	Europe	56.7	47.86666667	Mari	NA	Uralic	23
Mordovia	15	Europe	54.43333333	44.45	Mordvinic	NA	Uralic	30
North Italian	11	Europe	46	10	Italian	Italic	Indo-European	10
Orcadian	13	Europe	59	-3	NA	Germanic	Indo-European	10
Romania	16	Europe	44.41666667	26.1	Romanian	Italic	Indo-European	16
Romania Jew	3	Europe	44.41666667	26.1	NA	NA	NA	16
Russian	24	Europe	61	40	Russian	Balto-Slavic	Indo-European	10
Sardinian	27	Europe	40	9	Italian	Italic	Indo-European	10
Slovenian	25	Europe	46.05	14.5	Slovene	Balto-Slavic	Indo-European	36
Spain	12	Europe	40.43333333	-3.7	Spanish	Italic	Indo-European	16
TSI	107	Europe	43.78333333	11.25	Italian	Italic	Indo-European	31
Tuscan	6	Europe	43	11	Italian	Italic	Indo-European	10
Ukraine	20	Europe	49	32	Ukranian	Balto-Slavic	Indo-European	30
Bedouin	45	Middle East	31	35	Arabic	Semitic	Afroasiatic	10
Cyprus	12	Middle East	35	33	NA	NA	NA	16
Druze	41	Middle East	32	35	Arabic	Semitic	Afroasiatic	10
Iran	19	Middle East	32	53	Iranian	Indo-Iranian	Indo-European	16
Iran Jew	4	Middle East	32	53	NA	NA	NA	16
Iraq Jew	10	Middle East	33	44	NA	NA	NA	16
Jordan	20	Middle East	31.95	35.93333333	Arabic	Semitic	Afroasiatic	16
Kurd2	24	Middle East	37	43	Kurdish	Indo-Iranian	Indo-European	36
Lebanon	75	Middle East	33.9	35.53333333	Arabic	Semitic	Afroasiatic	35
Lebanon2	7	Middle East	33.9	35.53333333	Arabic	Semitic	Afroasiatic	16
Palestinian	44	Middle East	32	35	Arabic	Semitic	Afroasiatic	10

Qatari	16	Middle East	25.28666667	51.53333333	Arabic	Semitic	Afroasiatic	20
Samaritan	2	Middle East	32.06666667	34.78333333	Aramaic	Semitic	Afroasiatic	16
Saudi Arabia	20	Middle East	24	45	Arabic	Semitic	Afroasiatic	16
Syria	16	Middle East	33.5	36.3	Arabic	Semitic	Afroasiatic	16
Turkey	19	Middle East	40	30	Turkish	Oghuz	Turkic	16
Turkey Jew	10	Middle East	40	30	NA	NA	NA	16
Yemen	8	Middle East	15.34833333	44.20638889	Arabic	Semitic	Afroasiatic	16
Yemen Jew	15	Middle East	15.34833333	44.20638889	NA	NA	NA	16
Melanesian	5	Oceania	-6	155	Melanesian	NA	Austronesian	10
Papuan	2	Oceania	-4	143	NA	NA	Papuan	10

[1] Positive values indicate north and negative values indicate south.

[2] Positive values indicate east and negative values indicate west.

Table S2. Ancestral components and proxy samples

Ancestral component	Exemplar[1]	Proportion[2]
Amerindian	Colombian/Surui/Karitiana/PEL	100.0%
Arabian	Qatari	85.0%
Central African	Mbuti Pygmy	100.0%
Circumpolar	EastGreenland	93.7%
Eastern African	Anuak	70.4%
Japanese	JPT	91.0%
Kalash	Kalash	100.0%
Northern African	Tunisia	92.4%
Northen Asian	Yakut2	88.8%
Northern European	FIN	78.7%
Oceanian	Papuan/Melanesian	100.0%
Omotic	AriBlacksmith	97.3%
Sino-Tibetan	TTR_Tibetan	88.9%
South Indian	Nihali	81.7%
Southeastern Asian	Cambodian	80.3%
Southern African	Karretjie	100.0%
Southern Asian	Brahui	53.5%
Southern European	Basque2	76.4%
West-Central African	ESN	66.3%
Western African	Mandenka	89.3%
Western Asian	Abkhasian	56.8%

[1] The sample with the highest proportion of the given ancestral component is designated as the Exemplar.

[2] The percentage of the ancestral component in the exemplar is the Proportion. The data are denoised and renormalized.

Table S3. Average percentage of ancestral component by sample after denoising and renormalization, with exemplars hightlighted

Sample	Northern Asian	West-Central African	Omotic	Northern European	Japanese	Eastern African	Kalash	Northern African	Sino-Tibetan	Circum-polar	Amer-indian	Southern Asian	Western Asian	Central African	South-eastern Asian	South Indian	Southern European	Southern African	Oceanian	Western African	Arabian
Abkhasian	0	0	0	0.1104	0	0	0.0320	0	0	0.0070	0	0.2322	0.5685	0	0	0	0.0205	0	0	0	0.0294
ACB	0	0.5234	0.0111	0.0051	0	0.0022	0	0	0	0	0	0.0025	0.0028	0.0286	0	0	0.0091	0.0128	0	0.4025	0
Adygei	0.0343	0	0	0.1796	0	0	0.0299	0	0	0	0.0059	0.2231	0.4372	0	0	0	0.0661	0	0.0090	0	0.0149
Agaw	0	0	0.2013	0	0	0.3245	0	0.0805	0	0	0	0	0.0468	0	0	0	0	0	0	0	0.3469
Algeria	0	0.0395	0	0.0120	0	0.0168	0	0.4390	0	0	0	0.0199	0.0828	0	0	0	0.1739	0	0	0.0469	0.1692
Altaian	0.4083	0	0	0.1048	0.1243	0	0.0409	0	0.1656	0.0312	0.0326	0.0779	0	0	0	0	0.0145	0	0	0	0
Altaian2	0.3533	0	0	0.3121	0	0	0.0944	0	0.0568	0	0.0512	0.1091	0	0	0	0	0	0	0.0232	0	0
amaXhosa	0	0.5394	0	0	0	0.0728	0	0	0	0	0	0	0	0.0583	0	0	0	0.2428	0	0.0867	0
Amhara	0	0	0.2602	0	0	0.2546	0	0.0847	0	0	0	0	0.0410	0	0	0	0	0	0	0	0.3595
Andalusia	0	0	0	0.1863	0	0	0.0140	0.0766	0	0	0	0.0392	0.1339	0	0	0	0.4918	0	0.0090	0	0.0493
Anuak	0	0.0828	0.0662	0	0	0.7036	0	0	0	0	0	0	0	0.0914	0	0	0	0	0	0.0559	0
AriBlacksmith	0	0	0.9731	0	0	0	0	0	0	0	0	0.0119	0	0.0150	0	0	0	0	0	0	0
AriCultivator	0	0	0.7270	0	0	0.1358	0	0	0	0	0	0	0	0.0267	0	0	0	0.0289	0	0	0.0817
Armenia	0	0	0	0.0573	0	0	0.0316	0.0170	0	0.0051	0	0.1855	0.4845	0	0	0.0079	0.1009	0	0.0047	0	0.1055
Armenia2	0	0	0	0.0288	0	0	0.0333	0	0	0.0052	0	0.2030	0.4850	0	0	0	0.0882	0	0.0098	0	0.1466
Arunthathiyar	0	0	0	0.0193	0	0	0	0	0.0321	0	0	0.1958	0.0354	0	0	0.6797	0	0	0.0377	0	0
AshkenaziE	0	0	0	0.1217	0	0	0	0.0685	0	0	0	0.0954	0.3335	0	0	0	0.2413	0	0.0100	0	0.1296
AshkenaziW	0	0	0	0.0851	0	0	0	0.0647	0	0	0	0.0952	0.3294	0	0	0	0.2970	0	0	0	0.1286
Asur	0	0	0	0	0	0	0	0	0	0.0472	0	0	0	0	0.1851	0.6868	0	0	0.0809	0	0
ASW	0	0.0925	0.0342	0.0777	0	0.0235	0	0	0	0	0.0031	0.0212	0.0174	0.1143	0	0	0.1992	0.0466	0	0.3703	0
Athabask	0.0178	0	0	0	0	0	0	0	0	0.3396	0.6426	0	0	0	0	0	0	0	0	0	0
AzerbaijanJ	0	0	0	0.0341	0	0	0.0324	0	0	0	0.0070	0.1712	0.4699	0	0	0.0083	0.0882	0	0	0	0.1888
Balkar	0.0294	0	0	0.1856	0.0091	0	0.0192	0	0.0137	0	0.0046	0.2187	0.4427	0	0	0	0.0554	0	0.0054	0	0.0161
Balochi	0	0	0	0.0444	0	0	0.0336	0.0065	0	0.0103	0	0.4877	0.1316	0	0	0.1834	0.0143	0	0.0049	0	0.0835
Bamoun	0	0.5865	0	0	0	0.0794	0	0	0	0	0	0	0	0.0972	0	0	0	0.0124	0	0.2245	0
BantuKenya	0	0.5176	0	0	0	0.2672	0	0	0	0	0	0	0	0.1162	0	0	0	0.0238	0	0.0751	0
BantuSouthAfrica	0	0.5746	0	0	0	0.0701	0	0	0	0	0	0	0	0.0724	0	0	0	0.2049	0	0.0780	0
Basque1	0	0	0	0.1286	0	0	0.0287	0	0	0	0	0.0689	0.0174	0	0	0	0.7317	0	0.0075	0	0.0171
Basque2	0	0	0	0.1206	0	0	0.0297	0	0	0	0	0.0855	0	0	0	0	0.7641	0	0	0	0
Basters	0	0	0	0.1984	0	0.0053	0.0149	0.0060	0	0	0	0.0471	0.0416	0	0.0220	0.0550	0.2212	0.3754	0.0067	0	0.0064
BEB	0.0061	0	0	0.0373	0.0040	0	0.0210	0	0.0447	0.0089	0.0071	0.2378	0.0064	0	0.0416	0.5666	0.0068	0	0.0117	0	0
Bedouin	0	0	0	0.0055	0	0	0.0152	0.0263	0	0	0	0.0942	0.2157	0	0	0	0.0333	0	0	0	0.6098
Belorussia	0	0	0	0.5760	0	0	0.0297	0	0	0	0	0.0227	0.1121	0	0	0	0.2406	0	0	0	0.0188
Bengali	0	0	0	0	0.1009	0	0	0	0	0	0	0.2610	0	0	0	0.5885	0	0	0.0496	0	0
Bhunjia	0	0	0	0	0	0	0	0	0	0	0	0	0	0	0.1729	0.7531	0	0	0.0740	0	0
BiakaPygmy	0	0.1963	0	0	0	0	0	0	0	0	0	0	0	0.7844	0	0	0	0	0	0.0193	0
Bonda	0.0524	0	0	0	0	0	0	0	0	0	0	0	0	0	0.2925	0.5633	0	0	0.0918	0	0
Brahui	0	0	0	0.0238	0	0	0.0367	0.0113	0	0.0122	0	0.5349	0.1102	0	0.0036	0.1510	0.0260	0	0	0	0.0904
Brong	0	0.4643	0.0192	0	0	0.0154	0	0	0	0	0	0	0	0.0198	0	0	0	0.0091	0	0.4722	0
Bulala	0	0.1735	0	0	0	0.5789	0	0.0056	0	0	0	0	0	0.0210	0	0	0	0	0	0.2107	0.0102
Bulgaria	0	0	0	0.2958	0	0	0.0138	0.0140	0	0.0102	0	0.0495	0.2606	0	0	0	0.2873	0	0	0	0.0688
BulgariaJ	0	0	0	0.0965	0	0	0.0238	0.0747	0	0	0	0.0671	0.3310	0	0	0	0.2533	0	0	0	0.1535
Burmese	0.0261	0	0	0	0.0468	0	0	0	0.3859	0.0078	0	0	0	0	0.3418	0.1441	0	0	0.0473	0	0
Burusho	0.0119	0	0	0.1083	0	0	0.0831	0	0.0811	0	0.0218	0.3411	0.0940	0	0	0.2476	0	0	0.0111	0	0
Buryat	0.5177	0	0	0.0095	0.1700	0	0	0	0.2088	0.0213	0.0062	0.0460	0	0	0.0097	0	0	0	0.0110	0	0
Buryat2	0.4816	0	0	0.0098	0.2011	0	0	0	0.2213	0.0180	0.0090	0.0196	0.0112	0	0.0214	0	0	0	0.0069	0	0
Cambodian	0	0	0	0	0	0	0	0	0.0271	0	0	0	0	0	0.8030	0.0949	0	0	0.0750	0	0
CanaryIslands	0	0	0	0.1748	0	0	0.0120	0.1436	0	0	0.0098	0.0471	0.1169	0	0	0.0045	0.4184	0	0	0	0.0728
CDX	0.0055	0	0	0	0.1594	0	0	0	0.0491	0.0016	0	0	0	0	0.7607	0.0012	0	0	0.0226	0	0
CEU	0	0	0	0.3883	0	0	0.0292	0.0021	0	0.0029	0	0.0784	0.0863	0	0	0.0012	0.4089	0	0	0	0.0026
Chamar	0	0	0	0.0093	0	0	0.0196	0	0.0137	0	0.0132	0.2034	0	0	0	0.7266	0	0	0.0142	0	0
CHB	0.0019	0	0	0	0.4038	0	0	0	0.3004	0	0	0	0	0	0.2939	0	0	0	0	0	0
Chechen	0.0112	0	0	0.2127	0	0	0.0566	0	0	0.0067	0.0100	0.2289	0.4266	0	0	0	0.0169	0	0	0	0.0303
Chenchu	0	0	0	0	0.0362	0	0.0405	0	0	0	0	0.2114	0	0	0	0.6741	0	0	0.0377	0	0
CHS	0	0	0	0	0.3485	0	0	0	0.2098	0	0	0	0	0	0.4417	0	0	0	0	0	0
Chukchi	0.2321	0	0	0	0	0	0	0	0	0.7679	0	0	0	0	0	0	0	0	0	0	0
Chukchi2	0	0	0	0.5892	0	0	0	0	0	0	0	0	0.1200	0	0	0	0.2909	0	0	0	0
Chuvash	0.1674	0	0	0.4904	0	0	0.0153	0	0	0.0415	0.0067	0.0760	0.0744	0	0	0	0.1086	0	0.0057	0	0.0139
CLM	0	0	0	0.0924	0	0	0.0093	0.0258	0	0.0039	0.3256	0.0366	0.0805	0	0	0	0.3898	0	0.0033	0	0.0328
Colombian	0	0	0	0	0	0	0	0	0	0	1	0	0	0	0	0	0	0	0	0	0
ColouredColesberg	0	0.2830	0	0.0044	0	0	0	0	0	0	0	0.0111	0	0.0252	0	0.0232	0.0180	0.6101	0	0.0250	0
ColouredD6	0	0.1484	0	0.1392	0	0	0.0176	0	0	0	0	0.0664	0.0338	0	0.1142	0.1840	0.1030	0.1343	0.0592	0	0
ColouredEC	0	0.2811	0	0.0314	0	0.0623	0	0	0	0	0	0.0515	0	0	0	0.0424	0.0305	0.4863	0.0146	0	0
ColouredNC	0	0	0.0153	0.1491	0	0	0	0	0	0	0	0.0415	0	0	0.0157	0.0737	0.1891	0.4996	0	0	0.0159
ColouredWellingto	0	0.1765	0.0155	0.1198	0	0.0217	0.0212	0	0.0109	0	0	0.0552	0.0207	0	0.0721	0.1532	0.0467	0.2240	0.0300	0.0172	0.0152
Cyprus	0	0	0	0.0162	0	0	0	0.0497	0	0	0	0.1036	0.4592	0	0	0	0.1868	0	0	0	0.1844
Dai	0	0	0	0	0.1482	0	0	0	0.0388	0	0	0	0	0	0.7822	0.0066	0	0	0.0243	0	0
Daur	0.3005	0	0	0	0.3523	0	0	0	0.2471	0.0108	0.0064	0	0	0	0.0829	0	0	0	0	0	0
Dharkar	0	0	0	0.0555	0	0	0.0416	0	0.0097	0	0.0087	0.2745	0.0323	0	0	0.5456	0.0114	0	0.0206	0	0
Dhurwa	0	0	0	0	0	0	0	0	0	0	0	0	0	0	0.2456	0.6783	0	0	0.0761	0	0
Dolgan	0.7851	0	0	0.0390	0	0	0	0	0.0918	0.0222	0.0189	0	0.0228	0	0	0.0203	0	0	0	0	0
Dolgan2	0.8349	0	0	0	0	0	0	0	0.0759	0.0514	0	0	0	0	0.0378	0	0	0	0	0	0
Druze	0	0	0	0.0170	0	0	0.0164	0.0290	0	0	0	0.0873	0.5495	0	0	0.0024	0.0682	0	0.0058	0	0.2243
Dusadh	0	0	0	0.0182	0	0	0.0467	0	0	0.0199	0	0.2778	0	0	0	0.6067	0	0	0.0308	0	0
EastGreenland	0	0	0	0	0	0	0	0	0	0.9375	0.0625	0	0	0	0	0	0	0	0	0	0
EastGreenland2	0	0	0	0.2334	0	0	0	0	0	0.4779	0.0484	0.0439	0	0	0	0	0.1963	0	0	0	0
Egypt	0	0.0103	0.0130	0	0	0.0669	0	0.1443	0	0	0	0.0500	0.2855	0	0	0	0.1096	0	0	0	0.3202
Egypt2	0	0.0273	0.0224	0	0	0.0729	0	0.1335	0	0	0	0.0571	0.2861	0	0	0	0.0651	0	0	0.0082	0.3273
ESN	0	0.6629	0.0160	0	0	0.0066	0	0	0	0	0	0	0	0.0159	0	0	0	0.0049	0	0.2937	0
Estonia	0.0068	0	0	0.6629	0	0	0.0206	0	0	0	0	0.0499	0.0297	0	0	0.0062	0.2074	0	0	0	0.0165
EthiopiaJ	0	0	0.2442	0	0	0.2926	0	0.0950	0	0	0	0	0.0278	0	0	0	0	0	0	0.0103	0.3301
EthiopianAmhara	0	0	0.2449	0	0	0.2482	0	0.1168	0	0	0	0	0.0488	0	0	0	0	0	0	0	0.3413
EthiopianOromo	0	0	0.2694	0	0	0.3763	0	0.0629	0	0	0	0	0	0.0129	0	0	0	0.0201	0	0	0.2585
EthiopianSomali	0	0	0.2308	0	0	0.3812	0	0.0836	0	0	0	0	0	0	0	0	0	0	0	0	0.3044
EthiopianTigray	0	0	0.1949	0	0	0.2868	0	0.0933	0	0	0	0	0.0579	0	0	0	0	0	0	0	0.3671
Even	0.8580	0	0	0	0.0252	0	0	0	0	0.1167	0	0	0	0	0	0	0	0	0	0	0
Even2	0.7598	0	0	0	0.0773	0	0	0	0	0.1630	0	0	0	0	0	0	0	0	0	0	0
Evenki	0.8670	0	0	0	0.0205	0	0	0	0.0861	0	0.0136	0	0	0	0.0128	0	0	0	0	0	0
Fang	0	0.5669	0	0	0	0.0766	0	0	0	0	0	0	0	0.1509	0	0	0	0.0198	0	0.1858	0
FIN	0	0	0	0.7867	0	0	0.0068	0.0070	0	0.0088	0	0.0077	0.0157	0	0	0.0029	0.1291	0	0.0043	0	0.0310
French	0	0	0	0.3047	0	0	0.0225	0.0144	0	0.0036	0	0.0694	0.1242	0	0	0	0.4316	0	0.0069	0	0.0225
Gadaba	0	0	0	0	0	0	0	0	0	0	0	0	0	0	0.2673	0.6252	0	0	0.1075	0	0
Galicia	0	0	0	0.2076	0	0	0.0275	0.0916	0	0	0	0.0446	0.1141	0	0	0	0.4762	0	0	0	0.0383
Garo	0	0	0	0	0	0	0	0	0.4707	0	0	0	0	0	0.3139	0.1599	0	0	0.0556	0	0
GBR	0	0	0	0.3810	0	0	0.0315	0.0018	0	0	0.0020	0.0899	0.0567	0	0	0	0.4282	0	0.0056	0	0.0033
Georgia	0	0	0	0.1037	0	0	0.0473	0	0	0	0	0.2055	0.5580	0	0	0	0.0255	0	0.0044	0	0.0555

GeorgiaJ	0	0	0	0	0	0	0.0455	0.0584	0	0	0	0.1642	0.4453	0	0	0	0.0801	0	0	0	0.2064
GIH	0	0	0	0.0110	0	0	0.0409	0	0	0.0087	0.0016	0.3606	0.0115	0	0	0.5544	0	0	0.0066	0	0.0047
Gond	0	0	0	0	0	0	0	0	0	0	0	0.1578	0	0	0.0912	0.6946	0	0	0.0564	0	0
GuiGhanaKgal	0	0.1740	0	0	0	0	0	0	0	0	0	0	0	0.0410	0	0	0	0.7592	0	0.0258	0
Gumuz	0	0	0.3396	0	0	0.5601	0	0	0	0	0	0	0	0.0701	0	0	0	0	0.0065	0.0236	0
GWD	0	0.0500	0	0	0	0.0551	0	0.0007	0	0	0	0	0	0.0111	0	0	0	0.0068	0	0.8763	0
Hadza	0	0.0721	0.4175	0	0	0.1546	0	0	0	0	0	0	0	0.2669	0	0	0	0.0676	0	0.0213	0
Hakkipikki	0	0	0	0	0	0	0	0	0	0.0243	0	0.1960	0	0	0	0.7050	0.0246	0	0.0501	0	0
Han	0	0	0	0	0.3621	0	0	0	0.2069	0	0	0	0	0	0.4310	0	0	0	0	0	0
HanNChina	0.0253	0	0	0	0.3910	0	0	0	0.3550	0	0	0	0	0	0.2287	0	0	0	0	0	0
Hausa	0	0.5570	0	0	0	0.0890	0	0	0	0	0	0	0	0.0207	0	0	0	0.0087	0	0.3246	0
Hazara	0.1833	0	0	0.0873	0.0955	0	0.0225	0	0.1711	0	0.0153	0.1765	0.1147	0	0.0422	0.0599	0.0133	0	0.0081	0	0.0104
Hazara2	0.1311	0	0	0.1131	0.0518	0	0	0.0186	0.1177	0	0.0359	0.2693	0.1350	0	0	0.1078	0	0	0	0	0.0197
Hezhen	0.3240	0	0	0	0.4137	0	0	0	0.2381	0.0091	0	0	0	0	0.0150	0	0	0	0	0	0
Ho	0.0183	0	0	0	0	0	0	0	0	0	0	0	0	0	0.2303	0.6690	0	0	0.0823	0	0
Hungary	0	0	0	0.4143	0	0	0.0125	0	0	0	0	0.0676	0.1543	0	0	0	0.3241	0	0	0	0.0272
IBS	0	0	0	0.1972	0	0	0.0172	0.0389	0	0.0020	0	0.0494	0.1182	0	0	0	0.5250	0	0.0050	0	0.0470
Iran	0	0	0	0.0513	0	0	0.0394	0.0159	0	0	0	0.2799	0.3663	0	0	0.0330	0.0488	0	0.0060	0	0.1594
IranJ	0	0	0	0	0	0	0.0478	0.0305	0	0	0	0.2064	0.4136	0	0	0.0239	0.0717	0	0	0	0.2060
IraqJ	0	0	0	0	0	0	0.0344	0.0333	0	0	0	0.1903	0.4272	0	0	0	0.0776	0	0.0167	0	0.2205
ITU	0	0	0	0	0.0031	0	0.0275	0	0	0.0058	0.0026	0.2925	0.0174	0	0.0016	0.6221	0.0018	0	0.0206	0	0.0050
Japanese	0.0183	0	0	0	0.9065	0	0	0	0.0357	0	0	0	0	0	0.0243	0	0	0	0.0152	0	0
Jordan	0	0.0087	0.0069	0	0	0.0156	0.0125	0.0859	0	0	0	0.1499	0.3558	0	0	0	0.0907	0	0.0068	0	0.2672
JPT	0.0169	0	0	0	0.9096	0	0	0	0.0230	0.0023	0	0	0	0	0.0368	0	0	0	0.0114	0	0
Juang	0	0	0	0	0	0	0	0	0	0	0	0	0	0	0.3007	0.6095	0	0	0.0898	0	0
Juhoan	0	0	0	0	0	0	0	0	0	0	0	0	0	0.0576	0	0	0	0.9424	0	0	0
Juhoansi	0	0	0	0	0	0	0	0	0	0	0	0	0	0.0959	0	0	0	0.9041	0	0	0
Kaba	0	0.4479	0	0	0	0.1894	0	0	0	0	0	0	0	0.0515	0	0	0	0.0152	0	0.2960	0
Kalash	0	0	0	0	0	0	1	0	0	0	0	0	0	0	0	0	0	0	0	0	0
Kanjar	0	0	0	0.0812	0	0	0.0272	0	0.0230	0	0	0.3024	0	0	0	0.5662	0	0	0	0	0
Kanjars	0	0	0	0.0639	0	0	0.0695	0	0	0	0	0.2872	0	0	0	0.5794	0	0	0	0	0
Karitiana	0	0	0	0	0	0	0	0	0	0	1	0	0	0	0	0	0	0	0	0	0
Karretjie	0	0	0	0	0	0	0	0	0	0	0	0	0	0	0	0	0	1	0	0	0
Kazakhstan	0.2754	0	0	0.1431	0.1305	0	0.0239	0	0.1582	0.0324	0.0083	0.0772	0.0757	0	0.0322	0.0110	0.0321	0	0	0	0
Ket	0.5820	0	0	0.2172	0	0	0	0	0	0.1027	0.0417	0.0564	0	0	0	0	0	0	0	0	0
Ket2	0.5386	0	0	0.1966	0	0	0	0	0	0.1272	0.0402	0.0974	0	0	0	0	0	0	0	0	0
Khakas	0.2329	0	0	0.3066	0.0160	0	0.0378	0	0.1853	0.0139	0.0308	0.1408	0	0	0	0	0.0232	0	0.0128	0	0
Kharia	0	0	0	0	0	0	0	0	0	0	0	0	0	0	0.2641	0.6291	0	0	0.1068	0	0
Khasi	0.0466	0	0	0.0250	0	0	0	0	0.2439	0	0.0202	0.0298	0	0	0.2986	0.2673	0	0	0.0686	0	0
Khomani	0	0	0	0	0	0	0	0	0	0	0	0	0	0.0050	0	0	0	0.9950	0	0	0
Khomani2	0	0	0	0	0	0	0	0	0	0	0	0	0	0	0	0	0	1	0	0	0
KHV	0.0032	0	0	0	0.2049	0	0	0	0.0709	0.0016	0	0	0	0	0.7032	0	0	0	0.0161	0	0
Khwe	0	0.3630	0	0	0	0.0744	0	0	0	0	0	0	0	0.1250	0	0	0	0.3747	0	0.0572	0.0056
KochiJ	0	0	0	0.0350	0	0	0	0	0.0201	0.0177	0.0173	0.2953	0.0821	0	0	0.4409	0.0429	0	0	0	0.0487
Kol	0.0134	0	0	0.0405	0	0	0.0384	0	0.0111	0.0076	0	0.2427	0	0	0	0.6285	0	0	0.0177	0	0
Kongo	0	0.5999	0	0	0	0.0931	0	0	0	0	0	0	0	0.1175	0	0	0	0.0106	0	0.1789	0
Koryak	0.4175	0	0	0	0	0	0	0	0	0.5825	0	0	0	0	0	0	0	0	0	0	0
Koryak2	0	0	0	0.5823	0	0	0	0	0	0	0	0.0987	0	0	0	0	0.2656	0	0	0	0.0533
Kshatriya	0.0130	0	0	0.1097	0	0	0.0644	0	0	0.0105	0.0098	0.3203	0	0	0	0.4475	0.0248	0	0	0	0
Kumyk	0.0205	0	0	0.1755	0.0091	0	0.0319	0	0.0096	0	0	0.2225	0.4155	0	0	0	0.0627	0	0	0	0.0527
Kurd	0	0	0	0.0626	0	0	0.0475	0	0	0	0	0.2626	0.4149	0	0	0.0361	0.0422	0	0	0	0.1341
Kurd2	0	0	0	0.0503	0	0	0.0507	0.0147	0	0	0.0052	0.2804	0.3943	0	0	0.0127	0.0414	0	0.0049	0	0.1455
Kurmi	0	0	0	0.0723	0	0	0	0	0	0	0	0.3183	0	0	0	0.6094	0	0	0	0	0
Kurumba	0.0194	0	0	0	0	0	0	0	0	0	0	0.2688	0.0460	0	0	0.6430	0	0	0.0227	0	0
Kyrgyzstan	0.2962	0	0	0.1087	0.1734	0	0.0177	0	0.1913	0.0219	0.0195	0.0842	0.0513	0	0.0214	0.0057	0	0	0.0087	0	0
Kyrgyzstani	0.2908	0	0	0.1056	0.1614	0	0.0184	0	0.1955	0.0134	0.0088	0.0937	0.0526	0	0.0232	0	0.0162	0	0.0124	0	0.0081
Lahu	0.0251	0	0	0	0.0553	0	0	0	0.2771	0	0	0	0	0	0.6140	0.0062	0	0	0.0223	0	0
Lambadi	0	0	0	0.0632	0	0	0	0	0	0	0	0.2710	0.0976	0	0	0.5682	0	0	0	0	0
Lebanon	0	0	0	0.0078	0	0	0.0214	0.0501	0	0.0015	0	0.1262	0.4367	0	0	0.0053	0.1168	0	0.0071	0	0.2271
Lebanon2	0	0.0093	0	0	0	0.0119	0.0483	0.0571	0	0	0	0.1157	0.3969	0	0	0	0.1229	0	0	0	0.2379
Lezgian	0	0	0	0.2147	0	0	0.0369	0	0	0.0056	0	0.2833	0.4075	0	0	0	0.0318	0	0.0049	0	0.0152
Libya	0	0.0161	0.0110	0	0	0.0437	0.0132	0.2885	0	0	0	0.0294	0.1407	0.0068	0	0	0.1273	0	0.0060	0.0147	0.3026
Lithuania	0	0	0	0.6177	0	0	0.0152	0	0	0	0	0.0718	0.0518	0	0	0	0.2345	0	0.0090	0	0
LWK	0	0.5613	0.0072	0	0	0.2675	0	0	0	0	0	0	0	0.1255	0	0	0	0.0075	0	0.0309	0
Mada	0	0.2824	0	0	0	0.3708	0	0	0	0	0	0	0	0.0307	0	0	0	0	0	0.3162	0
Maduo_Tibetan	0.0342	0	0	0	0.0438	0	0	0	0.8504	0.0102	0	0	0	0	0.0174	0.0168	0	0	0.0272	0	0
Makrani	0	0.0034	0	0.0158	0	0	0.0421	0.0128	0	0	0	0.5142	0.1254	0	0	0.1571	0.0216	0	0	0	0.1076
Malaya	0.0544	0	0	0	0	0	0	0	0	0	0	0.1081	0	0	0	0.7394	0	0	0.0981	0	0
Mandenka	0	0.0421	0	0	0	0.0509	0	0	0	0	0	0	0	0	0	0	0	0.0141	0	0.8929	0
Mari	0.2400	0	0	0.4997	0	0	0.0219	0	0	0.0673	0	0.0675	0.0467	0	0	0	0.0456	0	0	0	0.0113
Mawasi	0	0	0	0	0	0	0	0	0	0	0	0	0	0	0.1773	0.7124	0	0	0.1103	0	0
Maya	0	0	0	0	0	0	0	0	0	0.0221	0.9779	0	0	0	0	0	0	0	0	0	0
MbutiPygmy	0	0	0	0	0	0	0	0	0	0	0	0	0	1	0	0	0	0	0	0	0
Meena	0	0	0	0	0	0	0	0	0	0	0.0398	0.3834	0	0	0	0.4858	0.0911	0	0	0	0
Meghawal	0.0668	0	0	0	0	0	0.0744	0	0	0	0	0.3015	0	0	0	0.5573	0	0	0	0	0
Melanesian	0	0	0	0	0	0	0	0	0	0	0	0	0	0	0	0	0	0	1	0	0
Miaozu	0	0	0	0	0.2597	0	0	0	0.2810	0	0	0	0	0	0.4593	0	0	0	0	0	0
MKK	0	0.1103	0.1317	0	0	0.5007	0	0.0241	0	0	0	0	0	0.0533	0	0	0	0.0321	0	0.0061	0.1418
Mongolia	0.2507	0	0	0	0.3560	0	0	0	0.2890	0	0	0	0	0	0.1043	0	0	0	0	0	0
Mongolia2	0.3820	0	0	0.0502	0.1886	0	0	0	0.2265	0.0317	0.0185	0.0399	0.0235	0	0.0390	0	0	0	0	0	0
Mordovia	0.0389	0	0	0.5636	0	0	0.0268	0	0	0.0164	0	0.0593	0.1054	0	0	0	0.1834	0	0.0063	0	0
Morocco	0	0.0494	0	0	0	0.0391	0	0.5091	0	0	0	0	0.0480	0.0090	0	0	0.1254	0	0	0.0812	0.1387
Morocco_N	0	0.0079	0.0137	0.0261	0	0.0077	0	0.4969	0	0	0	0.0214	0.0681	0	0	0	0.1787	0	0.0113	0.0373	0.1309
Morocco_S	0	0.0172	0.0133	0	0	0.0442	0	0.5292	0	0	0	0	0	0	0	0	0.1141	0	0	0.1807	0.1013
MoroccoJ	0	0	0	0.0600	0	0	0.0247	0.1289	0	0.0081	0	0.0668	0.3092	0	0	0	0.2413	0	0	0	0.1610
Mozabite	0	0.0272	0	0.0307	0	0.0270	0	0.7041	0	0	0	0.0210	0.0297	0.0059	0	0	0.0558	0	0	0.0348	0.0639
MSL	0	0.2509	0.0064	0	0	0.0029	0	0	0	0	0	0	0	0.0574	0	0	0	0.0253	0	0.6570	0
MumbaiJ	0	0	0	0	0	0	0	0	0	0	0	0.2394	0.2744	0	0	0.3641	0	0	0.0335	0	0.0886
Muslim	0	0	0	0.0707	0	0	0.0576	0	0.0274	0.0138	0	0.2525	0	0	0	0.5780	0	0	0	0	0
MXL	0	0	0	0.0137	0	0	0.0039	0	0	0.0117	0.7245	0.0248	0.0087	0	0	0	0.2064	0	0	0	0.0063
Naga	0	0	0	0	0.1675	0	0	0	0.6841	0	0	0	0	0	0.1180	0	0	0	0.0305	0	0
Nama	0	0	0.0224	0	0	0.0362	0	0	0	0	0	0	0	0	0	0	0	0.9183	0	0	0.0231
Naxi	0.0177	0	0	0	0.1546	0	0	0	0.5669	0.0127	0	0	0	0	0.2173	0.0075	0	0	0.0234	0	0
Nganassan	0.8643	0	0	0.0434	0	0	0	0	0	0.0923	0	0	0	0	0	0	0	0	0	0	0
Nihali	0	0	0	0	0	0	0	0	0	0	0	0.0534	0	0	0.0847	0.8174	0	0	0.0444	0	0
Nivkh	0.3411	0	0	0	0.4914	0	0	0	0	0.1352	0	0	0	0	0	0	0	0	0.0323	0	0
Nogai	0.1015	0	0	0.1977	0.0694	0	0.0405	0	0.0417	0.0112	0.0068	0.1520	0.2885	0	0	0	0.0650	0	0.0060	0	0.0197
NorthItalian	0	0	0	0.1901	0	0	0.0196	0.0166	0	0	0	0.0517	0.2414	0	0	0	0.4140	0	0	0	0.0667
NorthKannadi	0	0	0	0	0	0	0	0	0.0306	0.0365	0	0.1758	0	0	0	0.7123	0	0	0.0448	0	0

NorthOssetia	0.0441	0	0	0.1538	0	0	0.0244	0	0.0122	0.0108	0	0.2232	0.4919	0	0	0	0.0296	0	0	0	0.0100
Orcadian	0	0	0	0.4130	0	0	0.0436	0	0	0	0	0.0889	0.0406	0	0	0	0.4062	0	0	0	0.0078
Oromo	0	0	0.3096	0	0	0.2824	0	0.0749	0	0	0	0	0.0306	0	0	0	0	0.0111	0	0	0.2913
Oroqen	0.4465	0	0	0	0.2901	0	0	0	0.1725	0.0244	0	0	0	0	0.0664	0	0	0	0	0	0
Palestinian	0	0	0	0.0093	0	0.0064	0.0123	0.0732	0	0	0	0.1152	0.3776	0	0	0.0048	0.0869	0	0.0053	0	0.3090
Paniya	0	0	0	0	0.0676	0	0	0	0	0	0	0	0	0	0.5972	0.0748	0	0	0.2603	0	0
Papuan	0	0	0	0	0	0	0	0	0	0	0	0	0	0	0	0	0	0	1	0	0
Pashtun	0.0177	0	0	0.1464	0	0	0.0450	0	0.0573	0	0.0127	0.3584	0.1339	0	0	0.1280	0.0364	0	0.0320	0	0.0322
Pathan	0.0082	0	0	0.1204	0	0	0.0570	0	0	0.0085	0.0103	0.3659	0.1465	0	0	0.2552	0.0076	0	0	0	0.0204
PEL	0	0	0	0	0	0	0	0	0	0	1	0	0	0	0	0	0	0	0	0	0
Pima	0	0	0	0	0	0	0	0	0	0.0784	0.9216	0	0	0	0	0	0	0	0	0	0
PiramalaiKallars	0.0183	0	0	0	0	0	0	0	0	0	0	0.2575	0.0511	0	0	0.6478	0	0	0.0254	0	0
PJL	0.0041	0	0	0.0678	0	0	0.0448	0	0.0022	0.0052	0.0085	0.3278	0.0386	0	0	0.4739	0.0116	0	0.0091	0	0.0064
Pulliyar	0	0	0	0	0	0	0.0234	0	0	0	0.0129	0	0.0932	0	0	0.8057	0	0	0.0648	0	0
PUR	0	0.0291	0	0.1156	0	0	0.0140	0.0653	0	0.0041	0.1707	0.0387	0.0807	0	0	0.0019	0.4233	0	0.0032	0.0203	0.0331
Qatari	0	0	0	0.0246	0	0	0	0	0	0	0	0.1075	0	0	0	0	0.0176	0	0	0	0.8502
QH_Mongolian	0.2864	0	0	0.0147	0.1927	0	0.0093	0	0.4073	0.0163	0.0053	0.0183	0.0048	0	0.0376	0	0	0	0.0073	0	0
Romania	0	0	0	0.2793	0	0	0.0125	0.0159	0	0	0.0044	0.0893	0.2588	0	0	0.0051	0.2713	0	0	0	0.0634
RomaniaJ	0	0	0	0.1410	0	0	0	0.0301	0	0	0	0.0485	0.3556	0	0	0	0.2830	0	0	0	0.1418
Russian	0.0487	0	0	0.5937	0	0	0.0205	0	0	0.0081	0.0077	0.0550	0.0615	0	0	0	0.1906	0	0	0	0.0141
Sahrawi	0	0	0	0	0	0.0273	0	0.5923	0	0	0	0	0	0.0052	0	0.0121	0.0805	0	0	0.0990	0.1836
Samaritan	0	0	0	0	0	0	0	0.0516	0	0	0	0.0982	0.4355	0	0	0	0.1243	0	0	0	0.2904
San	0	0	0	0	0	0	0	0	0	0	0	0	0	0.0895	0	0	0	0.9105	0	0	0
Sandawe	0	0.2243	0.2347	0	0	0.2208	0	0.0088	0	0	0	0	0	0.1027	0	0	0	0.0991	0	0	0.1097
Santhal	0	0	0	0	0	0	0	0	0.0804	0	0	0	0	0	0.1174	0.7013	0	0	0.1009	0	0
Sardinian	0	0	0	0.0081	0	0	0	0.0568	0	0	0	0	0.2375	0	0	0	0.6238	0	0	0	0.0737
SaudiArabia	0	0	0	0.0181	0	0.0060	0.0136	0.0462	0	0	0.0042	0.1154	0.2553	0	0	0	0	0	0.0051	0	0.5361
Savara	0	0	0	0.0273	0	0	0	0	0	0	0	0	0	0	0.2265	0.6437	0	0	0.1024	0	0
SEBantu	0	0.5571	0	0	0	0.0684	0	0	0	0	0	0	0	0.0556	0	0	0	0.2422	0	0.0768	0
Selkup	0.5358	0	0	0.2655	0	0	0.0180	0	0	0.0972	0.0288	0.0547	0	0	0	0	0	0	0	0	0
Selkup2	0.6526	0	0	0.2024	0	0	0	0	0	0.0851	0.0222	0.0375	0	0	0	0	0	0	0	0	0
SGVP_CHS	0	0	0	0	0.3493	0	0	0	0.1805	0	0	0	0	0	0.4702	0	0	0	0	0	0
SGVP_INS	0.0032	0	0	0.0043	0	0	0.0344	0	0	0.0065	0	0.3030	0.0307	0	0.0049	0.5812	0.0042	0	0.0194	0	0.0081
SGVP_MAS	0.0214	0	0	0	0	0	0	0	0	0.0063	0	0	0	0	0.7718	0.0942	0	0	0.1063	0	0
She	0	0	0	0	0.3403	0	0	0	0.2083	0	0	0	0	0	0.4514	0	0	0	0	0	0
Shor	0.3645	0	0	0.1980	0.0815	0	0.0521	0	0.1163	0.0387	0.0359	0.1131	0	0	0	0	0	0	0	0	0
Sindhi	0	0	0	0.0709	0	0	0.0480	0	0	0.0061	0.0086	0.4113	0.0982	0	0	0.3129	0.0087	0	0.0102	0	0.0252
Slovenian	0	0	0	0.4291	0	0	0.0134	0	0	0	0	0.0537	0.1604	0	0	0	0.3167	0	0.0050	0	0.0218
Somali	0	0	0.1901	0	0	0.4233	0	0.0868	0	0	0	0	0	0	0	0	0	0	0	0	0.2998
Spain	0	0	0	0.2022	0	0	0.0248	0.0440	0	0	0	0.0414	0.1453	0	0	0	0.5116	0	0	0	0.0306
STU	0.0021	0	0	0	0	0	0.0118	0	0.0054	0.0103	0.0044	0.2813	0.0175	0	0	0.6257	0	0	0.0326	0	0.0088
Sudanese	0	0.1229	0.0192	0	0	0.7019	0	0	0	0	0	0	0	0.0879	0	0	0	0.0072	0	0.0610	0
Surui	0	0	0	0	0	0	0	0	0	0	1	0	0	0	0	0	0	0	0	0	0
SWBantu	0	0.5832	0	0	0	0.0788	0	0	0	0	0	0	0	0.1026	0	0	0	0.1027	0	0.1328	0
Syria	0	0	0	0	0	0	0.0193	0.0454	0	0	0	0.1751	0.3751	0	0	0	0.1257	0	0	0	0.2594
Tajikistan	0.0982	0	0	0.1182	0.0333	0	0.0315	0	0.0513	0	0.0226	0.2791	0.2308	0	0	0.1147	0	0	0.0203	0	0
Tajikistan2	0.0709	0	0	0.1580	0.0257	0	0.0713	0	0.0258	0.0116	0.0199	0.2879	0.1935	0	0	0.0696	0.0512	0	0	0	0.0146
TamilNadu	0	0	0	0	0	0	0	0	0	0.0253	0	0.2931	0	0	0.0337	0.6479	0	0	0	0	0
Tharu	0	0	0	0.0623	0	0	0.0483	0	0.0507	0	0	0.3033	0	0	0	0.5354	0	0	0	0	0
TN_Brahmin	0	0	0	0.0512	0	0	0	0	0.0534	0	0	0.3736	0	0	0	0.5218	0	0	0	0	0
TSI	0	0	0	0.1655	0	0	0.0151	0.0239	0	0.0017	0.0008	0.0553	0.2817	0	0	0	0.3681	0	0.0055	0	0.0824
TTR_Tibetan	0.0239	0	0	0	0	0	0	0	0.8893	0.0151	0.0028	0	0	0	0.0110	0.0248	0	0	0.0331	0	0
Tu	0.0474	0	0	0	0.2382	0	0	0	0.5287	0	0	0.0123	0	0	0.1734	0	0	0	0	0	0
Tujia	0	0	0	0	0.3129	0	0	0	0.2920	0	0	0	0	0	0.3951	0	0	0	0	0	0
Tunisia	0	0	0	0	0	0	0.0124	0.9237	0	0	0	0.0152	0.0259	0	0	0	0.0166	0.0062	0	0	0
Turkey	0.0327	0	0	0.0972	0	0	0.0256	0.0179	0	0.0088	0.0080	0.1813	0.3915	0	0	0.0076	0.1201	0	0.0047	0	0.1047
TurkeyJ	0	0	0	0.0673	0	0	0.0185	0.0924	0	0	0	0.0810	0.3573	0	0	0	0.2098	0	0	0	0.1737
Turkmenistan	0.1578	0	0	0.1386	0	0	0.0437	0	0.1190	0.0433	0	0.1862	0.1558	0	0	0.0341	0.0821	0	0	0	0.0393
Turkmenistan2	0.0732	0	0	0.1163	0.0346	0	0.0345	0.0136	0.0167	0	0.0166	0.2528	0.2718	0	0.0244	0.0519	0.0451	0	0	0	0.0483
Tuscan	0	0	0	0.1583	0	0	0.0269	0	0	0	0	0.0572	0.2891	0	0	0	0.3852	0	0	0	0.0833
Tuvinian	0.5114	0	0	0.0585	0.1413	0	0	0	0.1820	0.0472	0.0059	0.0333	0	0	0.0139	0	0	0	0.0065	0	0
Tygray	0	0	0.2127	0	0	0.2771	0	0.0834	0	0	0	0	0.0646	0	0	0	0	0	0	0	0.3623
Ukraine	0	0	0	0.5128	0	0	0.0122	0	0	0	0.0048	0.0527	0.1261	0	0	0.0064	0.2601	0	0.0051	0	0.0198
UP_Brahmin	0	0	0	0.1092	0	0	0.0453	0	0	0	0.0187	0.2943	0.0522	0	0	0.4382	0.0209	0	0.0213	0	0
UP_caste	0	0	0	0.0399	0.0238	0	0.0586	0	0	0	0	0.2306	0	0	0	0.6239	0	0	0.0232	0	0
Uttaranchal	0	0	0	0.0574	0.0818	0	0	0	0.2515	0	0.0392	0.2120	0	0	0	0.3580	0	0	0	0	0
Uygur	0.1445	0	0	0.1026	0.1449	0	0.0332	0	0.1615	0.0209	0	0.1467	0.0974	0	0.0557	0.0436	0.0368	0	0.0122	0	0
Uzbekistan	0.1686	0	0	0.1547	0.0748	0	0.0382	0.0104	0.1058	0.0260	0.0093	0.1603	0.1535	0	0.0149	0.0446	0.0307	0	0.0084	0	0
Uzbekistan2	0.0664	0	0	0.1393	0.0347	0	0.0267	0	0.0894	0	0	0.2776	0.1965	0	0	0.1310	0	0	0	0	0.0383
Uzbekistan3	0.1353	0	0	0.1370	0.0465	0	0.0245	0	0.0770	0	0.0126	0.2025	0.1860	0	0.0520	0.0694	0.0573	0	0	0	0
UzbekistanJ	0	0	0	0.0658	0	0	0.0721	0	0	0	0	0.1663	0.4058	0	0	0.0242	0.0757	0	0	0	0.1901
Velama	0	0	0	0	0	0	0.0245	0	0	0	0.0065	0.3041	0.0460	0	0	0.5798	0	0	0.0285	0	0.0106
WestGreenland	0	0	0	0.1015	0	0	0	0	0	0.7484	0.0687	0	0	0	0	0	0.0813	0	0	0	0
WestGreenland2	0	0	0	0.1237	0	0	0	0	0	0.6278	0.0776	0	0	0	0	0	0.1709	0	0	0	0
Wolayta	0	0	0.4550	0	0	0.2400	0	0.0255	0	0	0	0	0.0256	0	0	0	0	0	0	0	0.2540
Xibo	0.1804	0	0	0	0.4242	0	0	0	0.2820	0	0	0	0	0	0.1134	0	0	0	0	0	0
Xun	0	0.1128	0	0	0	0.0108	0	0	0	0	0	0	0	0.1088	0	0	0	0.7512	0	0.0164	0
Xun2	0	0.1130	0	0	0	0.0169	0	0	0	0	0	0	0	0.0919	0	0	0	0.7515	0	0.0268	0
XunV	0	0.0849	0	0	0	0	0	0	0	0	0	0	0	0.1264	0	0	0	0.7178	0	0.0709	0
Yakut	0.8181	0	0	0	0.0425	0	0	0	0.1121	0	0.0064	0	0	0	0.0156	0	0	0	0.0054	0	0
Yakut2	0.8877	0	0	0	0	0	0	0	0.1123	0	0	0	0	0	0	0	0	0	0	0	0
Yemen	0	0.0152	0.0317	0	0	0.0607	0	0.0576	0	0	0	0.1095	0.2677	0	0	0.0227	0.0220	0	0	0	0.4129
YemenJ	0	0	0.0372	0	0	0	0	0.0579	0	0.0061	0	0.0585	0.3142	0	0	0	0.0310	0	0.0112	0	0.4840
Yizu	0	0	0	0	0.1486	0	0	0	0.5967	0	0	0	0	0	0.2458	0	0	0	0.0089	0	0
Yoruba	0	0.6309	0	0	0	0.0278	0	0	0	0	0	0	0	0.0090	0	0	0	0.0069	0	0.3253	0
YRI	0	0.6032	0.0057	0	0	0.0234	0	0	0	0	0	0	0	0.0063	0	0	0	0.0039	0	0.3574	0
Yukaghir	0.7896	0	0	0	0	0	0	0	0	0.2104	0	0	0	0	0	0	0	0	0	0	0

Table S4. Pairwise F_{ST} between ancestral components

	Northern Asian	West-Central African	Omotic	Northern European	Japanese	Eastern African	Kalash	Northern African	Sino-Tibetan	Circumpolar	Amerindian	Southern Asian	Western Asian	Central African	Southeastern Asian	South Indian	Southern European	Southern African	Oceanian	Western African
West-Central African	0.193																			
Omotic	0.179	0.080																		
Northern European	0.128	0.168	0.147																	
Japanese	0.051	0.182	0.168	0.126																
Eastern African	0.190	0.058	0.074	0.164	0.179															
Kalash	0.131	0.172	0.152	0.076	0.124	0.167														
Northern African	0.149	0.139	0.119	0.076	0.140	0.135	0.100													
Sino-Tibetan	0.060	0.184	0.170	0.127	0.032	0.180	0.125	0.143												
Circumpolar	0.095	0.208	0.195	0.140	0.090	0.206	0.143	0.164	0.097											
Amerindian	0.117	0.213	0.199	0.139	0.108	0.211	0.146	0.166	0.115	0.107										
Southern Asian	0.144	0.171	0.151	0.072	0.137	0.166	0.077	0.095	0.138	0.154	0.154									
Western Asian	0.142	0.163	0.137	0.057	0.134	0.156	0.076	0.059	0.136	0.158	0.158	0.072								
Central African	0.217	0.075	0.099	0.193	0.207	0.095	0.196	0.171	0.207	0.231	0.237	0.196	0.189							
Southeastern Asian	0.074	0.185	0.170	0.132	0.036	0.182	0.129	0.145	0.046	0.108	0.122	0.142	0.139	0.209						
South Indian	0.113	0.162	0.146	0.102	0.097	0.158	0.091	0.115	0.100	0.134	0.138	0.111	0.102	0.188	0.100					
Southern European	0.144	0.171	0.146	0.048	0.138	0.166	0.086	0.060	0.139	0.158	0.157	0.080	0.044	0.196	0.143	0.112				
Southern African	0.238	0.107	0.118	0.214	0.228	0.124	0.216	0.194	0.230	0.254	0.259	0.217	0.208	0.084	0.231	0.209	0.216			
Oceanian	0.163	0.216	0.203	0.171	0.144	0.214	0.169	0.178	0.150	0.184	0.193	0.177	0.176	0.238	0.149	0.145	0.179	0.260		
Western African	0.192	0.026	0.078	0.167	0.181	0.053	0.169	0.137	0.183	0.207	0.212	0.169	0.161	0.071	0.184	0.160	0.169	0.104	0.214	
Arabian	0.152	0.164	0.142	0.081	0.144	0.161	0.098	0.067	0.145	0.167	0.170	0.093	0.063	0.191	0.149	0.117	0.063	0.211	0.184	0.162

Table S5. Comparison of F_{ST} between previously and newly defined ancestries

Previous	Current	F_{ST}	Raw *P*-value	Adjusted *P*-value
Kalash	Kalash	-0.002	1	1
Pygmy	Central African	-0.002	1	1
Berber	Northern African	-0.001	1	1
Click Speaker	Southern African	-0.001	1	1
Japanese	Japanese	0.001	0.280	1
Oceanian	Oceanian	0.008	0.155	1
Omotic	Omotic	0.006	0.128	1
Northern European	Northern European	0.001	0.124	1
Nilo-Saharan	Eastern African	0.003	0.101	1
Native American	Amerindian	0.005	0.068	1
Arabian	Arabian	0.006	1.88E-02	0.338
Southern European	Southern European	0.003	1.79E-02	0.322
Niger-Congo	West-Central African	0.004	1.27E-02	0.228
Siberian	Northern Asian	0.011	7.06E-03	0.127
Southeast Asian	Southeastern Asian	0.010	5.88E-04	1.06E-02
Chinese	Sino-Tibetan	0.016	4.41E-06	7.94E-05
Levantine-Caucasian	Western Asian	0.014	2.96E-06	5.33E-05
Indian	South Indian	0.021	4.77E-07	8.59E-06

Table S6. Correlations among ancestries and languages

Ancestry	Language	Correlation	*P*-value
Amerindian	Tupian	0.561	4.94E-22
	Tupian + Arawakan	0.688	2.69E-36
	Tupian + Arawakan + Quechumaran	0.796	7.12E-56
	Tupian + Arawakan + Quechumaran + Mayan	0.888	2.20E-85
	Tupian + Arawakan + Quechumaran + Mayan + Uto-Aztecan (Amerind)	0.962	6.17E-142
	Tupian + Arawakan + Quechumaran + Mayan + Uto-Aztecan + Na-Dené	0.989	8.34E-205
	Tupian + Arawakan + Quechumaran + Mayan + Uto-Aztecan + Yeniseian	0.840	1.60E-67
Arabian	Cushitic	0.336	5.46E-08
	Arabic	0.656	5.05E-32
	Semitic	0.774	7.28E-51
	Semitic + Cushitic	0.851	5.89E-71
Central African		NA	NA
Circumpolar	Eskimo-Aleut	0.766	2.15E-49
	Chukotko-Kamchatkan	0.355	8.47E-09
	Eskimo-Aleut + Chukotko-Kamchatkan (Paleosiberian)	0.799	1.41E-56
	Eskimo-Aleut + Chukotko-Kamchatkan + Na-Dené	0.815	1.46E-60
	Eskimo-Aleut + Chukotko-Kamchatkan + Yeniseian	0.750	2.92E-46
Eastern African	Cushitic	0.417	7.17E-12
	Nilo-Saharan	0.715	2.39E-40
	Cushitic + Nilo-Saharan	0.818	2.46E-61
Japanese	Japonic	0.644	1.55E-30
Kalash		NA	NA
Northern African	Berber	0.946	1.48E-122
Northern Asian	Mongolic	0.243	1.07E-04
	Turkic	0.394	1.09E-10
	Tungusic	0.412	1.35E-11
	Mongolic + Turkic + Tungusic (Altaic)	0.617	1.53E-27
	Mongolic + Turkic + Tungusic + Yukaghir	0.653	1.17E-31
	Mongolic + Turkic + Tungusic + Yukaghir + Uralic	0.698	1.14E-37
	Mongolic + Turkic + Tungusic + Yukaghir + Samoyedic	0.738	3.88E-44
	Mongolic + Turkic + Tungusic + Yukaghir + Samoyedic + Mari	0.740	1.83E-44
	Mongolic + Turkic + Tungusic + Yukaghir + Samoyedic + Mari + Mordvinic	0.731	8.06E-43
	Mongolic + Turkic + Tungusic + Yukaghir + Samoyedic + Mari + Na-Dené	0.729	1.29E-42
	Mongolic + Turkic + Tungusic + Yukaghir + Samoyedic + Mari + Yeniseian	0.781	2.53E-52
Northern European	Germanic	0.208	9.34E-04
	Finno-Ugric	0.424	2.63E-12
	Balto-Slavic	0.474	2.36E-15
	Mordvinic	0.218	5.26E-04
	Finno-Ugric + Mordvinic	0.478	1.28E-15
	Germanic + Finno-Ugric + Balto-Slavic	0.634	1.88E-29
	Germanic + Finno-Ugric + Balto-Slavic + Mordvinic	0.672	4.67E-34
Oceanian	Austronesian	0.673	3.14E-34
	Papuan	0.673	3.14E-34
	Austronesian + Papuan	0.954	3.36E-131
Omotic	Omotic	0.777	1.40E-51
Sino-Tibetan	Sino-Tibetan	0.726	4.20E-42
	Sino-Tibetan + Monguor	0.764	7.20E-49
	Sino-Tibetan + Monguor + Mongolic	0.793	3.83E-55
South Indian	Dravidian	0.552	2.81E-21
	Munda	0.424	2.78E-12
	Nihali	0.184	3.62E-03
	Dravidian + Munda + Nihali	0.740	2.03E-44
Southeastern Asian	Tai-Kadai	0.465	9.29E-15
	Khmer	0.342	2.93E-08
	Vietic	0.297	1.83E-06
	Khasi	0.113	0.0761

	Khmer + Vietic	0.453	5.22E-14
	Khmer + Vietic + Khasi	0.436	5.64E-13
	Khmer + Vietic + Tai-Kadai	0.652	1.67E-31
	Khmer + Vietic + Tai-Kadai + Khasi	0.635	1.65E-29
	Hmong-Mien	0.261	3.09E-05
	Khmer + Vietic + Tai-Kadai + Hmong-Mien	0.686	5.36E-36
Southern African	Kwadi-Khoe	0.381	5.27E-10
	Tuu	0.568	1.08E-22
	Kx'a	0.665	3.51E-33
	Kwadi-Khoe + Tuu + Kx'a	0.960	4.78E-138
Southern Asian	Indo-Iranian	0.584	3.92E-24
	Dravidian	0.306	8.46E-07
	Indo-Iranian + Dravidian	0.678	7.96E-35
Southern European	Italic	0.616	1.93E-27
	Basque	0.478	1.34E-15
	Italic + Basque	0.764	6.34E-49
Western African	Mande	0.797	5.64E-56
West-Central African	Bantu	0.727	2.79E-42
	non-Bantu	0.503	2.21E-17
	Bantu + non-Bantu	0.895	2.00E-88
Western Asian	Northeast Caucasian	0.233	2.11E-04
	Northwest Caucasian	0.291	3.02E-06
	Kartvelian	0.232	2.26E-04
	Northeast Caucasian + Northwest Caucasian + Kartvelian (Ibero-Caucasian)	0.438	4.43E-13
	Northeast Caucasian + Northwest Caucasian + Kartvelian + Armenian	0.522	8.31E-19
